# Modelling human endurance: power laws vs critical power

**DOI:** 10.1007/s00421-023-05274-5

**Published:** 2023-08-10

**Authors:** Jonah P. Drake, Axel Finke, Richard A. Ferguson

**Affiliations:** 1 Department of Mathematical Sciences, School of Science, Loughborough University, Loughborough, LE11 3TU UK; 2 School of Sport, Exercise and Health Sciences, Loughborough University, Loughborough, LE11 3TU UK

**Keywords:** Power-duration relationship, Performance prediction, Hyperbolic, Critical speed, Critical velocity, Pacing

## Abstract

The *power–duration relationship* describes the time to exhaustion for exercise at different intensities. It is believed to be a “fundamental bioenergetic property of living systems” that this relationship is hyperbolic. Indeed, the *hyperbolic* (a.k.a. *critical-power*) model which formalises this belief is the dominant tool for describing and predicting high-intensity exercise performance, e.g. in cycling, running, rowing or swimming. However, the hyperbolic model is now the focus of a heated debate in the literature because it unrealistically represents efforts that are short (< 2 min) or long (> 15 min). We contribute to this debate by demonstrating that the power–duration relationship is more adequately represented by an alternative, *power-law* model. In particular, we show that the often-observed good fit of the hyperbolic model between 2 and 15 min should not be taken as proof that the power–duration relationship is hyperbolic. Rather, in this range, a hyperbolic function just happens to approximate a power law fairly well. We also prove mathematical results which suggest that the power-law model is a safer tool for pace selection than the hyperbolic model and that the former more naturally models fatigue than the latter.

**Supplementary Information:**

The online version contains supplementary material available at 10.1007/s00421-023-05274-5.

## Introduction

### The power–duration relationship

The *power–duration relationship* describes the time to exhaustion for exercise at different intensities. Knowledge of this relationship is crucial to athletes and coaches, e.g. for:

- *fitness assessment*—i.e. in order to inform training, athletes (and their coaches) want to quantify and track their fitness level;
- *performance prediction*—i.e. in order to select race and pace strategies, athletes want to predict their potential over distances or durations that may not have recently been performed. For instance,
  - runners want to know their best possible finish time in a marathon from only a recent half-marathon performance (i.e. without running the full distance);
  - cyclists want to know if they can sustain a particular power output for a given distance or duration (e.g. needed at the final climb of a race).

In this work, we focus on the endurance sports: running, cycling and rowing, although we stress that our results are not limited to these activities and may even be relevant in other settings, e.g. functional capacity testing in clinical populations.

Unfortunately, an individual’s power–duration relationship is unknown and must be estimated from available data with the help of mathematical models that formalise this relationship. A number of such models have been proposed in the literature. Amongst these are two which were both originally used to model world-record performances across different athletes (and even animals) but which are nowadays also used to describe the power–duration relationship *within* individual athletes.

**Fig. 1:**
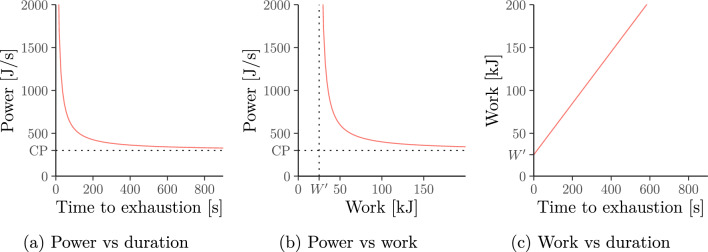
Equivalent relationships between power, duration and work under the hyperbolic (a.k.a. critical-power) model. Note that the power output approaches the critical power $\mathrm{CP}>0$ in Panels **a** and **b** as duration and work increase

**Fig. 2:**
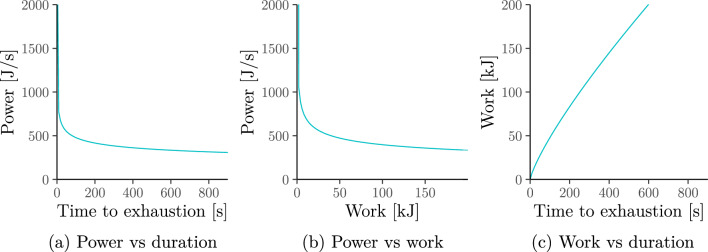
Equivalent relationships between power, duration and work under the power-law (a.k.a. Riegel) model. Note that the power output approaches $0$ in Panels **a** and **b** as duration and work increase. This is in contrast to the hyperbolic (a.k.a. critical-power) model in Fig. 1 where it approaches critical power $\mathrm{CP}>0$

- **Hyperbolic (a.k.a. critical power) model. **The *hyperbolic* model (Hill 1925, 1993; Monod and Scherrer 1965; Jones et al. 2010)–illustrated in Fig. 1–asserts that the power–duration relationship is hyperbolic.^1^ The power asymptote is called critical power. The model is thus also called the *critical-power* model.
- **Power-law (a.k.a. Riegel) model.** The *power–law* model (Kennelly 1906)–illustrated in Fig. 2–asserts that the power–duration relationship follows a power law. It was popularised in running by Riegel (1977, 1981) and is thus often termed the *Riegel* model.

### Recent debate about the critical-power paradigm

The hyperbolic (a.k.a. critical-power) model is thought to describe and predict endurance performance during high-intensity exercise (i.e. exercise classed as “severe”) “with startling precision” (Poole et al. 2016). It is ubiquitous in cycling (Leo et al. 2022a), where it is implemented in popular online exercise analytics platforms; but it is also widely used for training prescription and performance prediction in other endurance sports, such as running (Kranenburg and Smith 1996; Nimmerichter et al. 2017), rowing (Hill et al. 2003), swimming (Wakayoshi et al. 1992; Petrigna et al. 2022) as well as walking and skating (Hill 1925). Additionally, it has been applied to intermittent sports, such as football, hockey and rugby (Okuno et al. 2011) in order to optimise the length of recovery needed between exercise bouts (Fukuda et al. 2011). The model has also been suggested as a tool for anti-doping (Puchowicz et al. 2018) and for assessing the effectiveness of nutritional supplements (Fukuda et al. 2010; Stout et al. 1999). The hyperbolic model has even been employed to measure exercise capacity in horses and mice (Lauderdale and Hinchcliff 1999; Billat et al. 2005). Indeed, the hyperbolic shape of the power–duration relationship which the model formalises is considered to be a “fundamental bioenergetic property of living systems” (Jones et al. 2019).

However, in recent decades, doubts have been repeatedly raised about validity of the hyperbolicity assumption (see Dotan 2022a, and references therein). In particular, it is widely known that the model behaves unrealistically for exercise durations that are short (e.g. less than 2 min) or long (e.g. more than 15 min);

- over short durations, the hyperbolic model would imply that an elite runner like Eliud Kipchoge can sustain over 650 km/h over one second or instantaneously “teleport” over 175 m.
- over long durations, the hyperbolic model would imply that runners can complete ultra-marathons at essentially the same average velocity as half-marathons.

This debate about the hyperbolic model has recently intensified with the publication of the papers by Dotan (2022a), Gorostiaga et al. (2022b) and the ensuing discussions (Burnley 2022a, b; Black et al. 2022; Broxterman et al. 2022; Triska and Karsten 2022; Marwood and Goulding 2022; Altuna and Hopker 2022; Lindinger 2022; Abdalla et al. 2023; Dotan 2022b, c; Gorostiaga et al. 2022a, c, 2023).

### Contributions

We show that the power–duration relationship should not be presumed to be hyperbolic. Rather it is more adequately described by the power-law model; and that doing so resolves both the above-mentioned controversies about the critical-power paradigm.

Specifically, our novel contributions, both empirical and theoretical, are threefold:

- **Contribution I: Fit over different durations.** In the section: “Contribution I: Fit over different durations”, we compare the fit of the power-law and hyperbolic models in the context of (a) the case studies of elite athletes from Jones and Vanhatalo (2017), Jones et al. (2019) and (b) additional large data sets of runners, rowers and cyclists, and demonstrate that:
  - for exercise durations inside the 2–15 min range (for which the hyperbolic model is thought to be valid) the power-law model fits just as well as the hyperbolic model;
  - for exercise durations outside the 2–15 min range (for which the hyperbolic model predicts performances that are physically impossible), the power-law model still exhibits realistic behaviour.
  This suggests that the good performance of the hyperbolic model in the 2–15 min range cannot be taken as evidence for the hypothesis that the power–duration relationship is hyperbolic. Rather, the power–duration relationship is more adequately described by a power law and the hyperbolic model just happens to approximate the power-law model reasonably well in the 2–15 min range.
- **Contribution II: Implications for pacing.** In the section: “Contribution II: Implications for pacing”, we prove a mathematical result which suggests that the power-law model has more realistic implications about pacing than the hyperbolic model. That is, the power-law model implies that athletes will only achieve their best possible finish time in a time trial if they race at the highest constant velocity that they can sustain until the finish line; and over-pacing is always detrimental. In contrast, the hyperbolic model implies that the safest optimal pacing strategy is to sprint off as quickly as possible and then “hold on” until crossing the finish line. Thus, the power-law model is consistent with the pacing recommended by coaches and observed in elite athletes whereas the hyperbolic model is not.
- **Contribution III: Modelling fatigue.** In the section: “Contribution III: Modelling fatigue”, we demonstrate that, unlike the hyperbolic model, the power-law model is automatically consistent with the empirical observation that the power–duration curve moves downwards as the athlete becomes fatigued during prolonged exercise. We also explain why the often-made assumption that critical power decreases with fatigue is contradictory.

### Related literature

The finding that the power–duration relationship is more adequately modelled by a power law than a hyperbolic function has already been demonstrated in running (Hinckson and Hopkins 2005; Zinoubi et al. 2017; Girardi et al. 2022) and swimming (Osiecki et al. 2014).^2^ Even in cycling, where the hyperbolic model and variations thereof dominate the literature, Coakley (2015, Chapter 5) and Passfield et al. (2013) suggested that;

“[the] power law is better than the [critical-power] model for predicting cycling endurance performance across a wider range of exercise intensities.”

However, these studies do not appear to have had much impact on the above-mentioned debate surrounding the utility of the critical-power paradigm because they were purely empirical and based only on small sample sizes (between 8 and 15 individuals). In contrast, our evidence includes three large studies of 5805 cyclists, 2571 runners, and seasons’ best times from 3244 rowing ergometer sessions, as well as formal (i.e. mathematical) derivations.

## Power–duration models

Power–duration models are functions which assert a relationship between an exercise duration $T$ and a highest power $P\left(T\right)$ that can be sustained over that duration. Throughout this work, we focus mostly on this *power–duration* relationship $P=P\left(T\right)$. However, by simple algebra, this is always equivalent to, e.g. a particular *power–work* relationship $P=P\left(W\right)$ which characterises the highest constant power which the athlete can continuously generate until some work $W>0$ has been done; or to a particular *work–duration* relationship $W=W\left(T\right)$ which gives the maximum amount of work that can be performed over duration $T$. All of these relationships can be inverted, e.g. $T=T\left(P\right)$ is the time to exhaustion for some constant power output $P$.

Power metres are available on cycling and rowing ergometers and use of mobile power metres is now widespread in cycling, making it feasible to estimate and directly work with the power–duration relationship. However, for running, we will use “velocity” $\left(V\right)$ as a proxy for “power” $\left(P\right)$ and “distance” $\left(D\right)$ as a proxy for “work” $\left(W\right)$. This is commonly done in the literature and is justified if the conditions throughout the activity are relatively constant, e.g. flat course, no significant amount of wind and negligible air resistance/friction.

We stress that $P\left(T\right)$ (likewise $V\left(T\right)$) must be understood as the intensity that an athlete can *sustain* over a duration $T$ and does not model the initial acceleration (or the fatigue that occurs during the “acceleration phase”). Therefore, a cyclist’s observed maximal power output over one second will be below $P\left(T=1\mathrm{\ s}\right)$. Similarly, a runner’s observed average velocity in a 100-m sprint will be below $V\left(D=100\mathrm{\ m}\right)$. However, these effects become negligible over longer durations or distances.

### Hyperbolic (a.k.a. critical-power) model

#### The model

The *hyperbolic* model (Hill 1925; Monod and Scherrer 1965) (see also Jones et al. 2010, and references therein) assumes the following power–duration relationship which is also illustrated in Fig. 1a:

1 $$P={P}_{\mathrm{hyp}}(T)=\frac{{W}^{\prime}}{T}+\mathrm{CP},$$

for parameters ${W}^{\prime}, \mathrm{CP}>0$ and exercise durations $T>0$. That is, (1) posits that athletes can continuously generate power ${P}_{\mathrm{hyp}}\left(T\right)>\mathrm{CP}$ for at most $T$ seconds at which point they are exhausted. The parameters $\mathrm{CP}$ and ${W}^{\prime}$ are interpreted as follows.

- **Critical power** $\mathrm{CP}$**.** The additive constant $\mathrm{CP}>0$, called *critical power,* was originally interpreted as the power that could be sustained “for a very long time without fatigue” (Monod and Scherrer 1965). However, this interpretation is no longer meaningful as the relationship in (1) is nowadays assumed to only be valid for exercise durations $T$ that are not too long and not too short (we discuss this in the section “Range of validity” subsec ref).
- **Finite work capacity** ${W}^{\prime}$**.** Multiplying both sides of (1) by $T$ shows that athletes exercising at some constant power output $P>\mathrm{CP}$ will have generated a total amount of work equal to ${W}^{\prime}+T\cdot \mathrm{CP}$ by the time they reach exhaustion. Thus, the parameter ${W}^{\prime}$ is interpreted as representing a finite “tank” of exercise capacity “above $\mathrm{CP}$” available to the athlete. As discussed below, this interpretation still holds even if the power output $P>\mathrm{CP}$ is not constant.

Simple algebra implies﻿ that the power–duration relationship from (1) (Fig. 1a) can be equivalently written as a *power–work* relationship (Fig. 1b) and *work–duration* relationship (Fig. 1c). A full list of such equivalent relationships is given in Supplementary Information A.1.1. Finally, as discussed, we sometimes (e.g. in running) work with velocity $(V)$ and distance $(D)$ instead of power $(P)$ and work $(W)$. In this case, we write $D^{\prime}$ instead of $W^{\prime}$ and $\mathrm{CV}$ (*“critical velocity”*) instead of $\mathrm{CP}$.

#### Range of validity

The hyperbolic model, i.e. the relationship from (1), is nowadays assumed to only hold for exercise in the so-called “severe-intensity domain”, i.e. for exercise during which ${\dot{\text{V}}\text{O}}_{2} {\text{max}}$ is attained (Jones et al. 2019). More formally, the hyperbolic model is assumed to be valid for exercise durations $T$ such that ${P}_{\mathrm{L}}<{P}_{\mathrm{hyp}}\left(T\right)<{P}_{\mathrm{U}},$ where ${P}_{\mathrm{L}}$ and ${P}_{\mathrm{U}}$ are the lower and upper boundaries of the severe intensity domain. Unfortunately, the requirement that the model should apply only to “severe” exercise is impractical. This is because this requirement typically leaves the range of validity of the hyperbolic model (i.e. the durations $T$ or powers $P$ for which the model is applicable) ill-defined for the following reasons:

- **Unclear boundaries. **The boundaries ${P}_{\mathrm{L}}$ and ${P}_{\mathrm{U}}$ of the “severe” domain differ across individuals and are typically unknown. As a result, it is not always clear which data points (i.e. which power–duration measurements or race results) one can include or exclude when fitting the model or even for which race distances the model is allowed to make performance predictions. For instance, it is not clear whether the model applies to a 5-km run or a 20-min cycle ride.
- **Circular reasoning**. The requirement that the model should only be applied to “severe” exercise can also quickly become circular because the lower boundary of the severe domain, ${P}_{\mathrm{L}}$, is defined as being equal to the value of the parameter $\mathrm{CP}$ from the hyperbolic model, i.e. ${P}_{\mathrm{L}} :=\mathrm{CP}$. In other words, we should fit the hyperbolic model solely to exercise intensities above $\mathrm{CP}$ but we only know the value of $\mathrm{CP}$ *after* fitting the model to data. This circular reasoning is not merely a philosophical issue because the parameters ${W}^{\prime}$ and $\mathrm{CP}$ of the hyperbolic model are highly sensitive to the range of durations (and powers) of the data points used (Bishop et al. 1998; see also Dotan 2022a and references therein). In particular, as we include data points over longer and longer durations, the estimate of $\mathrm{CP}$ decreases. The only way to avoid this circular reasoning is to estimate ${P}_{\mathrm{L}}=\mathrm{CP}$ by other means but there is no simple alternative.^3^
- **Unbounded endurance.** Even if ${P}_{\mathrm{L}}$ and ${P}_{\mathrm{U}}$ were known exactly, assuming that the hyperbolic model is valid for all durations $T$ such that ${P}_{\mathrm{L}}<{P}_{\mathrm{hyp}}\left(T\right)<{P}_{\mathrm{U}}$ is still impractical. This is because the model would then formally imply that power outputs slightly above ${P}_{\mathrm{L}}=\mathrm{CP}$ could be sustained “for a very long time without fatigue”. For instance, the model would predict that a cyclist with ${P}_{\mathrm{L}}=\mathrm{CP}=300$ and ${W}^{\prime}=25,000$ would be able to sustain 300*.*1 J*/*s for over 70 h. However, this is no longer accepted in the literature (Vanhatalo et al. 2011; Poole et al. 2016).

For these reasons, we follow Jones et al. (2019) in assuming that the hyperbolic model is valid for exercise durations $T$ between 2 and 15 min. We note that the literature considers the 2–15 min range not as being strict but rather as being a “guide” in the sense that exercise durations up to 20 or even 25 min can sometimes still be included. However, throughout this work, we will use 15 min, whenever possible, because:

1. Knowing that the model can sometimes still work over durations longer than 15 min does not lead to a practically useful rule for deciding to which efforts the model can or cannot be applied.
2. None of our findings would change qualitatively if we chose, say, 2–20 or 2–25 min. In fact, our results in the section “Contribution I: Fit over different durations” suggest that 2–15 min is actually the best-case scenario for the hyperbolic model: its errors (compared with the power-law model) quickly increase outside this range.

#### Estimation

The parameters of the hyperbolic model are commonly estimated via linear regression by exploiting the fact that in (1), $P$ is (affine) linear in $1/T$. To keep the presentation simple, we adopt this approach throughout this work (except for some of the panels in Fig. 4). However, none of our results would change qualitatively if we used the more sophisticated non-linear weighted least-squares approaches (potentially based on the power–work relationship) advocated in Patoz et al. (2021). The estimation requires data from a sufficiently large number of maximal efforts (i.e. efforts to exhaustion) over different distances or durations with a time to exhaustion between 2 and 15 min (Karsten et al. 2015).

#### Rate-of-exertion interpretation

Solving (1) for $1/T$ as in Gordon (2005) implies that, under the hyperbolic model, the athlete’s *rate of exertion* when generating some power $P\ge \mathrm{CP}$ is given by

$${\text{rate}}_{{{\text{hyp}}}} \left( P \right): = \frac{{P - {\text{CP}}}}{{W^{\prime}}}.$$

Thus, the accumulated fatigue after exercise duration $t$ is:

2 $${\text{Fatigue}}_{t} : = \mathop \int \limits_{0}^{t} {\text{rate}}_{{{\text{hyp}}}} \left( {P\left\langle s \right\rangle } \right)~{\text{d}}s = \frac{{\mathop \int \nolimits_{0}^{t} \left( {{P\left\langle s \right\rangle } - {\text{CP}}} \right)~{\text{d}}s}}{{W^{\prime } }},$$

where $P\langle s\rangle \ge \mathrm{CP}$ denotes the instantaneous power output at time $s$. That is, at the start of the activity, the athlete is assumed to be rested (${\mathrm{Fatigue}}_{0}=0$). The athlete is exhausted (and has to drop power output down to at most $\mathrm{CP}$) at the first time $T$ such that ${\mathrm{Fatigue}}_{T}=100 \ \%$.

Equation 2 shows that exhaustion occurs when the amount of work generated “above” $\mathrm{CP}$, ${\int }_{0}^{T}\left(P\langle t\rangle - \mathrm{CP}\right)\, \mathrm{d}t$, equals ${W}^{\prime}$. Therefore, an often-used equivalent interpretation is that ${\text{Balance}}_{t} : = 1 - {\text{Fatigue}}_{t} \in \left[ {0,1} \right]$ is the remaining *balance* (measured as a proportion of ${W}^{\prime}$) of the athlete, i.e. athletes start with a full “${W}^{\prime}$ tank” (${\mathrm{Balance}}_{0}=1$) and they reach exhaustion at the first time $T$ such that they have fully emptied this tank (${\mathrm{Balance}}_{T}=0$).

### Power-law (a.k.a. Riegel) model

#### The model

The *power-law* model goes back to at least Kennelly (1906), Lietzke (1954) (see García-Manso et al. (2005), Vandewalle (2018) and references therein). It was popularised by Riegel (1977, 1981), especially in the long-distance running community where it implies a widely used rule-of-thumb for finishing-time prediction (see, e.g. Runner’s World Magazine 2013). It also forms the basis of the more sophisticated predictors from Blythe and Király (2016) and Vickers and Vertosick (2016). The latter is implemented by the websites Slate (Aschwanden 2014) and FiveThirtyEight (Aschwanden 2016).

The power-law model assumes the following power–duration relationship illustrated in Fig. 2a. For parameters $S>0$, $0<E<1$ and any duration $T>0$:

3 $$P={P}_{\mathrm{pow}}\left(T\right)=S{T}^{E-1}.$$

That is, (3) posits that athletes can continuously generate power ${P}_{\mathrm{pow}}\left(T\right)>0$ for at most $T$ seconds at which point they are exhausted.

**Fig. 3:**
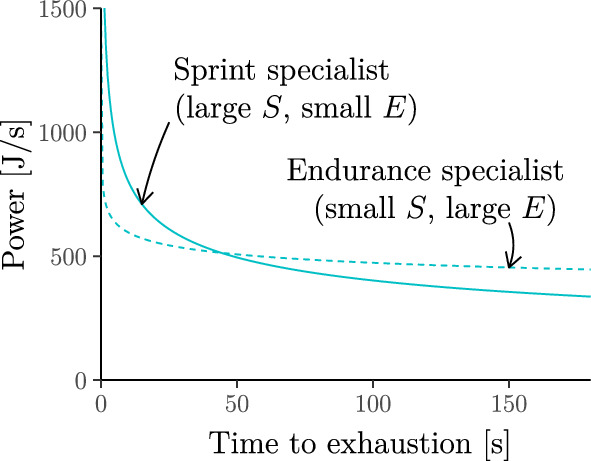
Role of the speed parameter $S$ and the endurance parameter $E$

As with the hyperbolic model, simple algebra allows us to convert (3) (Fig. 2a) into an equivalent power–work relationship (Fig. 2b) and work–duration relationship (Fig. 2c). A full list of such equivalent relationships is given in Supplementary Information A.2.1. The role of the parameters $S$ and $E$ are visualised in Fig. 3. Specifically:

- **Speed parameter** $S$**.** The *speed* parameter $S>0$ governs the vertical scaling of the power-duration curve. It is also the power that a cyclist can sustain for one second or the velocity that a runner can sustain for one second.
- **Endurance parameter** $E$**.** The *endurance* parameter $0<E<1$ governs how quickly the power–duration curve in Fig. 3 decays (smaller values of $E$ correspond to a quicker decay). Its reciprocal, $F=1/E>1$, was termed *fatigue factor* by Riegel (1981). Throughout this work, we will sometimes switch between $F$ and $E=1/F$ to simplify the presentation; Riegel (1981) also gave values for $F$ in different sports and different demographic groups (derived from world-record performances *across* different athletes, i.e. these may not be optimal for individual athletes).

The power-law model has been independently discovered many times in the literature. For instance, Hinckson and Hopkins (2005), Laursen et al. (2007) applied it to running but called it the *log–log* model because it implies affine–linear relationships between the log-duration or log-work and log-power; Pinot and Grappe (2011) found it to perform well in cycling.

Finally, García-Manso et al. (2012), Blythe and Király (2016) have noted that there are systematic patterns in the residuals when fitting the power-law model to world-record performances in running. However, we stress that this considers the power–duration (or velocity–duration) relationship averaged *across* different athletes. In contrast, our work is concerned with modelling this relationship *within* individual athletes and we agree with Broxterman et al. (2022) that “[d]etermining the speed–duration relationship across athletes (…) whilst interesting, is fundamentally different from determining this relationship within an athlete”. Indeed, Blythe and Király (2016) found that the power-law model performs better for the latter based on 1,417,432 performances of 164,746 runners (a subset of which we analyse in the next section), stating “(…) although world-record performances are known to obey a power law (…), there is no reason to suppose a priori that the performance of individuals is governed by a power law. Striking is that the power-law derived is considerably more accurate when considered in log-distance–log-speed coordinates than the power-law which applies to world-record data.”

#### Range of validity

As we will show in the next section, the power-law model describes exercise capacity well across a wide range of durations. In particular, unlike the hyperbolic model, the power-law model is not restricted to predicting only those power outputs that fall into a particular range (e.g. into a particular exercise-intensity domain). This makes the power-law model much easier to use and more widely applicable than the hyperbolic model. Of course, there are some obviously “silly” edge cases that should be avoided such as applying the model to durations $T$ longer than an individual’s lifespan. We also reiterate our comment from the section “Hyperbolic (a.k.a. critical-power) model” that observed performances over short efforts (e.g. 100 m sprints) will be below what the model predicts because the model describes the power or velocity that can be *sustained* over a particular duration or distance and thus does not account for the initial acceleration (whose impact in short races is substantial).

#### Estimation

The power-law model can again be easily estimated via linear regression by exploiting the fact that by (3), $\mathrm{log}(P)$ is (affine) linear in $\mathrm{log}(T)$. In this case, $\mathrm{exp}(intercept)$ is an estimate of $S$ and $(slope+1)$ is an estimate of $E$. To keep the presentation simple, we adopt this approach throughout this work. However, none of our results would change qualitatively if we used linear regression based on the fact that $\mathrm{log}(P)$ is (affine) linear in the log-work $\mathrm{log}(W)$ or if we used more sophisticated non-linear least-squares approaches such as those advocated for the hyperbolic model in Patoz et al. (2021).

Note that at least two data points must be available to estimate both model parameters. If only one data point is available, e.g. when predicting a marathon finish time from a single recent half-marathon result, it can be preferable to fix $E$ (equivalently: $F=1/E$) to a suitable default value. For instance, consider the popular calculator from Runner’s World Magazine (2013) which predicts the finish time $T$ in a race over distance $D$ from the finish time ${T}_{0}$ in a previous race over some other distance ${D}_{0}$ as

$$T={T}_{0}{\left(\frac{D}{{D}_{0}}\right)}^{1.06}\hspace{0.17em}.$$

This calculator is obtained from (3) by fixing $F=1.06$ (see Supplementary Information A.2.2 for details).

In other words, this calculator predicts the finish time using a power-law model in which the only the speed parameter, $S$, is estimated (from a single previous race result) whilst the endurance parameter is fixed to $F=1.06$.

Of course, the endurance parameter $E$ (equivalently: the fatigue factor $F$) is likely to be different for different athletes as shown in Blythe and Király (2016), Zinoubi et al. (2017) (otherwise, we could expect some athletes to simultaneously hold world records over both short and long race distances); and even for a given athlete, the parameter $E$ likely varies over time, e.g. with (de-)training (otherwise, faster 5-km run performance would automatically imply an improved marathon prediction). Hence, setting $E$ to a default value incurs a bias. Therefore, if sufficiently many data points (e.g. recent race results) are available, it is typically preferable to estimate both $S$ and $E$ (based on all data) rather than using methods like the calculator from Runner’s World Magazine (2013).

#### Rate-of-exertion interpretation

We end this section by showing that we can derive a similar “rate-of-exertion” interpretation for the power-law model as for the hyperbolic model. Solving (3) for $1/T$ implies that the athlete’s *rate of exertion* when generating some power $P\ge 0$ implied by the power-law model is given by

4 $${\text{rate}}_{{{\text{pow}}}} \left( P \right): = \left( \frac{P}{S} \right)^{{1/\left( {1 - E} \right)}} .$$

Thus, the accumulated fatigue after exercise duration $t$ is:

5 $${\text{Fatigue}}_{t} : = \mathop \int \limits_{0}^{t} {\text{rate}}_{{{\text{pow}}}} \left( {P\left\langle s \right\rangle } \right)~{\text{d}}s,$$

where $P\langle s\rangle \ge 0$ denotes the instantaneous power output at time $s$. That is, at the start of the activity, the athlete is assumed to be rested (${\mathrm{Fatigue}}_{0}=0$). The athlete is exhausted (and has to drop power output down to $0$) at the first time $T$ such that ${\mathrm{Fatigue}}_{T}=100 \ \%$.

Again, we can equivalently interpret ${\text{Balance}}_{t} : = 1 - {\text{Fatigue}}_{t} \in \left[ {0,1} \right]$ as a remaining “balance” (as a proportion of $1/{S}^{1/(1-E)}$) of the athlete. Importantly, (4) shows that this balance decreases faster than linearly in the power output, $P$. This contrasts with the hyperbolic model whose balance decreases only linearly in $P$ (more accurately: linearly in $P-\mathrm{CP}$). For this reason, no fixed work capacity (i.e. “${W}^{\prime}$-tank”) interpretation is possible for the power-law model.

To our knowledge, the rate-of-exertion interpretation of the power-law model is novel. In section “Contribution II: Implications for pacing”, we employ this construction to prove a mathematical statement which suggests that the power-law model has more realistic pacing implications than the hyperbolic model. In section “Contribution III: Modelling fatigue”, we employ this construction to show that, consistent with empirical evidence, the power–duration curve implied by the power-law model moves downwards as the athlete becomes more fatigued.

## Contribution I: Fit over different durations

It is well known that the hyperbolic model adequately describes exercise capacity over durations that are not too short and not too long, e.g. around 2–15 min, but exhibits unrealistic behaviour outside this range (see, e.g. Vandewalle et al. (1997); Jones et al. (2019); Pallarés et al. (2020)). In this section, we empirically show that the good fit of the hyperbolic model for exercise durations in the 2–15 min range does not mean that the power–duration relationship is hyperbolic. Rather, the power–duration relationship can be much better described by a power law and the hyperbolic model just happens to approximate the power-law model reasonably well in the 2–15 min range. We first demonstrate this result in the context of the case studies of elite runners from Jones et al. (2019), Jones and Vanhatalo (2017). We then show similar results in large-data studies of recreational runners, rowers and cyclists. Finally, we explain why the problems of the hyperbolic model become even more apparent when combining it with a different functional form over long durations, e.g. as done in the so-called *omni power–duration* models from Puchowicz et al. (2020).

### Case study from Jones et al. (2019)

Here, we reproduce the case study of Eliud Kipchoge from Jones et al. (2019). We fit the hyperbolic model to Eliud Kipchoge’s personal records for races in the 2–15 min range (${D}^{\prime}\approx 176.29 \ \mathrm{m}$, $\mathrm{CV} \approx 6.23 \ \mathrm{m}/\mathrm{s}$) and compare the results with the power-law model fitted to all of Eliud Kipchoge’s personal records in different events (including indoor events) up to marathon distance ($S \approx 9.57 \ \mathrm{m}/\mathrm{s}$, $E \approx 0.94$).^4^ The data was obtained from World Athletics (2021), however, for completeness, we provide Kipchoge’s personal records, e.g. 00:13:11 (5 km); 00:59:25 (half marathon) and 02:01:39 (marathon), in Supplementary Information B.2.

**Fig. 4:**
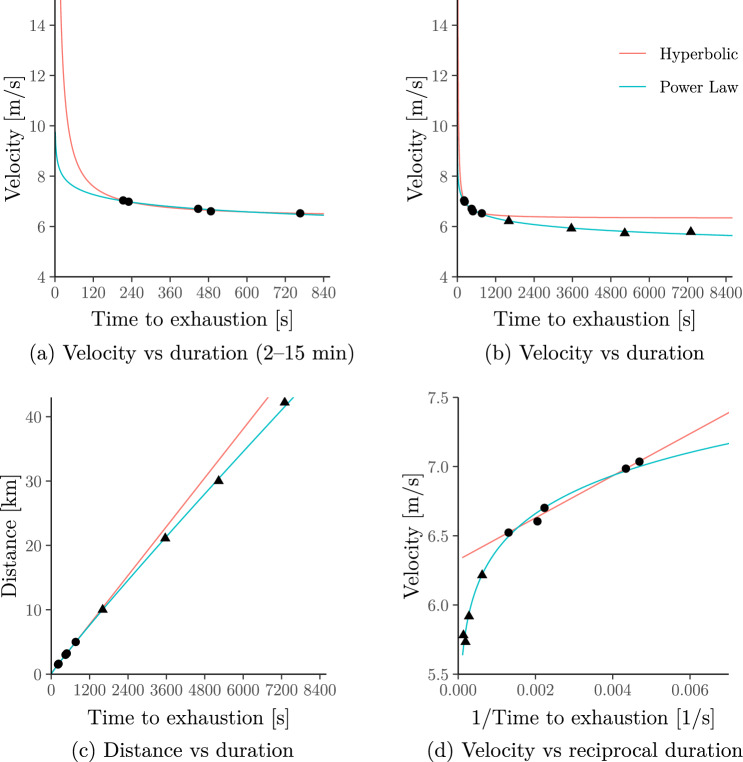
An extended and modified version of Jones et al. (2019, Fig. 5, Panels **a**–**d**) showing the hyperbolic (a.k.a. critical-power) model fitted to Eliud Kipchoge’s personal records. As in Jones et al. (2019), the hyperbolic model is fitted only to the personal records in the 2–15 min range (filled circles); the power-law (a.k.a. Riegel) model is fitted to all personal records up to marathon distance (filled circles and filled rectangles). Note that the power-law model behaves similarly to the hyperbolic model inside the 2–15 min range but exhibits more realistic behaviour outside this range

The results are shown in Fig. 4 which replicates all four panels from Jones et al. (2019, Fig. 5). It illustrates that, for Eliud Kipchoge’s data, the hyperbolic model predicts unrealistically high velocities for durations shorter than 2 min or longer than 15 min, whereas the power-law model still predicts realistic velocities over a wide range of durations:

- **Short durations.** If we were to apply the hyperbolic model to shorter exercise durations, it would imply that Eliud Kipchoge can
  - sustain more than 650 km*/*h over 1 s (85 km*/*h over 10 s)^5^;
  - instantaneously “teleport” over ${D}^{\prime}\approx 176$ metres (see the non-zero intercept in Fig. 1c; Proposition 3 in Supplementary Information B.1 gives a formal proof);
  In contrast, the power-law model implies that Eliud Kipchoge can
  - only sustain *less than* 35 km*/*h over 1 s (30 km*/*h over 10 s)—well below the top speeds of elite sprinters;
  - *not* cover any non-zero distance instantaneously (see the zero intercept Fig. 2c; Proposition 4 in Supplementary Information B.1 gives a formal proof).

- **Long durations.** If we were to apply the hyperbolic model to longer exercise durations, it would imply that Eliud Kipchoge can run ultra-marathons at essentially the same average velocity as half-marathons. In contrast, the power-law model implies that the velocity which Eliud Kipchoge can sustain over some duration/distance goes to zero as the duration/distance increases.

**Fig. 5:**
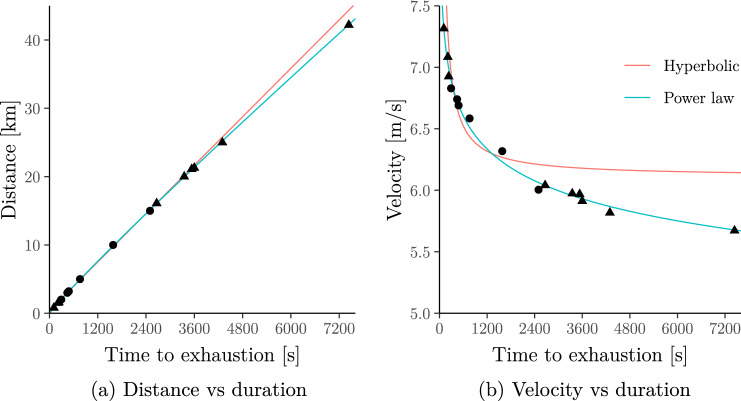
Haile Gebrselassie’s personal records in different events. Figure. 5a, extends Jones and Vanhatalo (2017, Fig. 2) to include races shorter than 1500 m and longer than 15,000 m. As in Jones and Vanhatalo (2017) the hyperbolic (a.k.a. critical-power) model is only fitted to the personal records in the 1500–15,000 m range (filled circles); the power-law (a.k.a. Riegel) model is fitted to all personal records up to marathon distance (filled circles and filled rectangles). The figure shows that the power-law model exhibits realistic behaviour over all durations up to marathon distance

Interestingly, in Fig. 4, the hyperbolic and power-law models give very similar behaviour for exercise durations in the 2–15 min range. Later in this section, we show that this holds for wider populations.

### Case study from Jones and Vanhatalo (2017)

We now reproduce the case study from Jones and Vanhatalo (2017), i.e. we fit the hyperbolic model to the personal records over different events (including indoor events) up to marathon distance of Haile Gebrselassie and 11 other elite runners. The data were again obtained from World Athletics (2021). We apply the hyperbolic model to all races with distances between 1500 m and 15,000 m which corresponds to exercise durations of around 3*.*6–41*.*6 min for Haile Gebrselassie. Clearly, this does not match the 2–15 min exercise duration range for which the hyperbolic model is thought to be valid but is done to match the analysis carried out in Jones and Vanhatalo (2017). We compare the results with the power-law model (fitted to all personal records up to marathon distance).^6^ For completeness, we provide Gebrselassie’s personal records, e.g. 00:12:39 (5000 m); 00:58:55 (half marathon) and 02:03:59 (marathon), in Supplementary Information B.3.

The first panel in Fig. 5 extends Jones and Vanhatalo (2017, Fig. 2) and shows that the power-law model fits well for Haile Gebrselassie’s personal records over different durations. For clarity, we have added the second panel which shows the same results but using the velocity–duration relationship.

**Fig. 6:**
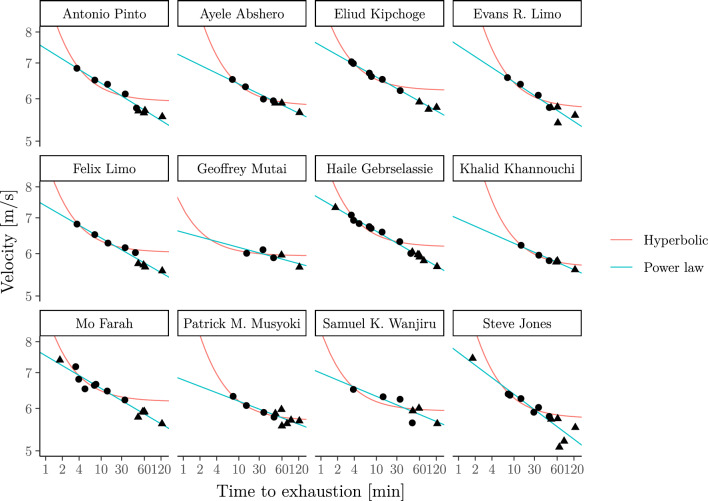
Personal records of the elite runners considered in Jones and Vanhatalo (2017, Table 1). As in Jones and Vanhatalo (2017), the hyperbolic (a.k.a. critical-power) model is only fitted to data in the 1500–15,000 m range (filled circles); the power-law model (a.k.a. Riegel) is fitted to all personal records up to marathon distance (filled circles and rectangles). Note that both axes are on the log-scale for clarity. The figure again shows that the power-law model exhibits realistic behaviour over all durations up to marathon distance (see Table 3 in Appendix B.3 for a more formal comparison of the fit of each model)

Figure 6 compares the fit of both models for the group of 12 male elite athletes (which include Eliud Kipchoge and Haile Gebrselassie) considered in Jones and Vanhatalo (2017, Table 1). A more formal numerical comparison of the errors of both models given in Table 3 in Supplementary Information B.3.

### Large-data study in running

**Fig. 7:**
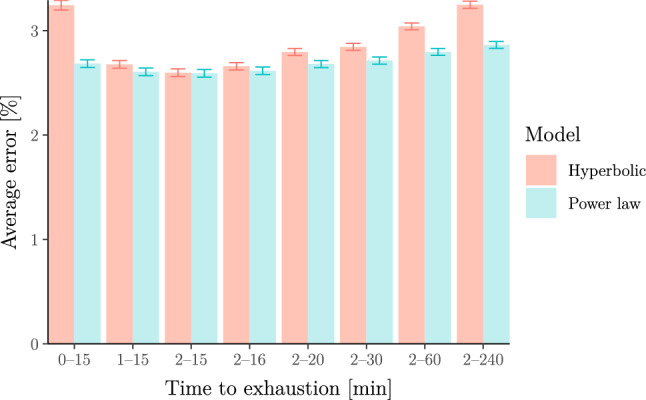
Relative error of the hyperbolic (a.k.a. critical-power) model vs the power-law (a.k.a. Riegel) model for 2571 runners. Errors are calculated as observed vs predicted relative velocities. The error bars represent standard errors. The figure illustrates that both models provide a similar fit for exercise durations in the 2–15 min range but that the power-law model provides a better fit outside this range

In the remainder of this section, we show that the above findings generalise to wider populations. First, we fit both models to race results from (mostly recreational) runners collected by Blythe and Király (2016) from the database powerof10.info. After removing athletes with too few records in the 2–15 min range (see Supplementary Information B.5 for details), we are left with race results for 2571 runners. For each of these, we fit both the hyperbolic and power-law models and compare the average error across all athletes, where error is defined as the average relative difference between observed and predicted velocities as in Puchowicz et al. (2020) (see Supplementary Information B.4). Figure 7 shows this error averaged across all athletes for each model. This error is similar for both models for exercise durations between 2 and 15 min but worse for the hyperbolic model if we include efforts shorter than 2 min or longer than 15 min.

### Large-data study in rowing

**Fig. 8:**
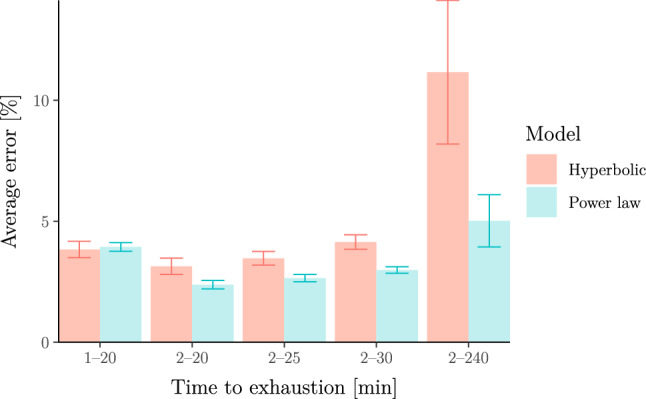
Relative error of the hyperbolic (a.k.a. critical-power) model vs the power-law (a.k.a. Riegel) model for 3244 athlete seasons in rowing. The errors and error bars are calculated as in Fig. 7. The figure again illustrates that the power-law model describes the data more adequately than the hyperbolic model over a wide range of durations longer than 2 min (note that results for 1–20 min are inconclusive due to the overlapping error bars)

Next, we fit both models to rowing-ergometer data available from www.nonathlon.com. Specifically, the data contain self-reported seasons’ bests (for seasons from 2002 to 2022) in the form of finish times over fixed distances 500 m, 1 km, 5 km, 6 km, 10 km, 21*.*0975 km (half marathon) and 42*.*195 km (marathon) as well as distances covered over fixed durations 30 min and 60 min. In total, we were left with 3244 athlete seasons after removing all athlete seasons with less than three measurements between 2 and 20 min (see Supplementary Information B.6 for details). We stress that we selected 20 min instead of 15 min as the minimum upper bound on the duration ranges because (a) only 506 athletes in the data set have at least three efforts in the 2–15 min range; (b) none of these have efforts shorter than 2 min; (c) all of these are “slow” athletes whose time over 500 m is above 2 min (i.e. focussing only on these athletes might incur a selection bias). We again fit both models to the data. The results are shown in Fig. 8 which again illustrates that the power-law model describes the data better than the hyperbolic model over a wide range of durations.

### Large-data study in cycling

**Fig. 9:**
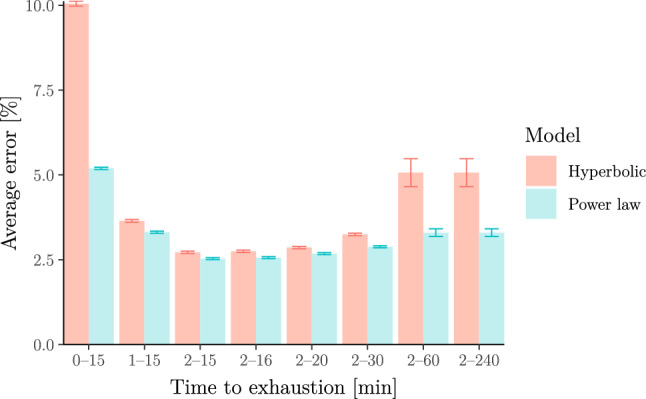
Relative error of the hyperbolic (a.k.a. critical-power) model vs the power-law (a.k.a. Riegel) model for 5805 cyclists. Errors are calculated as observed vs predicted relative powers. The error bars represent standard errors. The figure shows that errors are similar for exercise durations in the 2–15 min range but that the power-law model has smaller errors than the hyperbolic model outside this range

We now show (in Fig. 9) the same analysis as in the section “Large-data study in running” but applied to an open data set of 5805 cyclists from Golden Cheetah (www.goldencheetah.org). For each athlete, we extracted the best average power output for a range of different durations from their training and racing history. It is well known that it is difficult to fit these models on racing and training data as it is unclear whether the data corresponds to truly maximal efforts (Puchowicz et al. 2018; Leo et al. 2021, 2022b). To alleviate this, we removed some efforts that could not have been maximal as well as outliers which are likely to be due to power-metre malfunctions (see Supplementary Information B.7 for more details). The results shown in Fig. 9 again illustrate that the power-law model describes the data better than the hyperbolic model over a wide range of durations.

### Piecewise-defined models

**Fig. 10:**
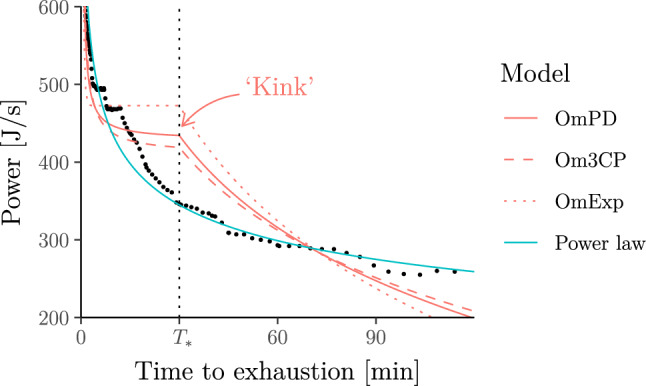
Power–duration relationship of the so-called omni power–duration models (OmPD, Om3CP and OmExp; with the same threshold ${T}_{*}=30$ min) from Puchowicz et al. (2020) for the data from Leo et al. (2022a, Fig. 4) (filled circles)

To permit more realistic predictions outside the 2–15 min range without abandoning the critical-power paradigm, it has been proposed to combine (variants of) the hyperbolic model for durations shorter than some threshold ${T}_{*}>0$ with different functional forms for durations longer than ${T}_{*}$ (Péronnet and Thibault 1989; Puchowicz et al. 2020; Luttikholt and Jones 2022) (see Fig. 10, and Supplementary Information B.8 for a more formal treatment). However, such “piecewise-defined” models introduce other drawbacks:

- They require an increased number of unknown parameters so that a large number of data points (e.g. from arduous critical-power tests or previous races) are needed to avoid overfitting—and a number of these data points must be from efforts longer than ${T}_{*}$. Additionally, the threshold ${T}_{*}$ is difficult to choose but its choice affects the other model parameters, e.g. $\mathrm{CP}$ and ${W}^{\prime}$ (Puchowicz et al. 2020).
- They posit a power–duration relationship that is actually no longer hyperbolic for exercise intensities above $\mathrm{CP}$ (or above a similar threshold), i.e. they are not compatible with the assumption that athletes have a fixed amount of work, ${W}^{\prime}$, that they can generate above $\mathrm{CP}$.
- They imply a power–duration relationship with unrealistic properties:
  - For sufficiently long durations, the power–duration curve from Péronnet and Thibault (1989), Puchowicz et al. (2020) can become negative.
  - The models from Péronnet and Thibault (1989), Puchowicz et al. (2020), Luttikholt and Jones (2022) have a “kink” in the power curve as shown in Fig. 10.

The final point connects to our findings from earlier in this section, that the hyperbolic model fits power–duration (or similar) data reasonably well for exercise durations $T$ which are not too short (e.g. at least 2 min) and not too long (e.g. at most ${T}_{*}=15$ min). However, as we saw, this is only because the hyperbolic function approximates a power law reasonably well in this range. Indeed, the fact that the hyperbolic assumption is problematic becomes immediately apparent in Fig. 10. Here, the need for reconciling the posited hyperbolic shape over durations ${T\le T}_{*}$ with the fact that the power–duration curve should approach zero as $T$ increases (as no exercise intensity can be maintained “for a long time without fatigue”) causes a “kink”.

Whilst this kink could be easily smoothed out, this would still leave a power–duration curve with turning/inflection points.^7^ Indeed, unless ${T}_{*}$ is trivially small, *any* model which combines a hyperbolic curve (below ${T}_{*}$) with another curve that decays to zero for long durations (above ${T}_{*}$) must exhibit such a kink, turning/inflection point or discontinuity. To our knowledge, there is no empirical evidence for the existence of such artefacts^8^ and this again calls the “hyperbolic” assumption into question.

**Fig. 11:**
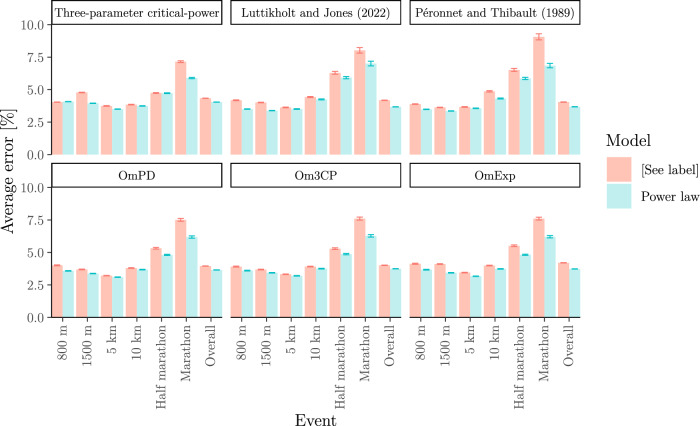
Out-of-sample relative predictive error for each piecewise-defined model vs the power-law model for the running dataset from the section  "Large-data study in running". For each athlete, we fitted the models to the same six randomly chosen race results and predicted average velocities of the remaining races. Note that the piecewise-defined models often could not be fitted as they require a certain minimum number of data points above and below ${T}_{*}$. Whenever this happened, we excluded the corresponding athlete to ensure that both models in each panel are applied to exactly the same data. This figure illustrates that the piecewise-defined models discussed in this section suffer from overfitting (at least for the small sample sizes that are typically available)

We end this section by comparing, in Fig. 11, the out-of-sample prediction error of the power-law model with the out-of-sample prediction error of all of the above mentioned piecewise-defined models, and additionally the *three-parameter critical-power* model (Morton 1996).

## Contribution II: Implications for pacing

In this section, we show that the power-law (a.k.a. Riegel) model has realistic pacing implications in the sense that it implies that over-pacing (e.g. running or riding off too quickly) is detrimental to the overall finish time in a race. This is in contrast to the hyperbolic (a.k.a. critical-power) model which implies that the safest optimal pacing strategy is to sprint off as fast as possible and then “hold on”.

Throughout this section, we assume that we want to generate some power $W$ over the shortest possible duration. Consider the following constant (“even”) pacing and variable (“uneven”) pacing strategies^9^:

- $\mathbf{P}\mathbf{a}\mathbf{c}{\mathbf{e}}_{\mathbf{c}\mathbf{o}\mathbf{n}}$ Generate power $P>0$ continuously over duration $T>0$ such that exhaustion occurs at time $T$ at which point work $W$ has been accumulated, i.e. $T\cdot P=W$.
- $\mathbf{P}\mathbf{a}\mathbf{c}{\mathbf{e}}_{\mathbf{v}\mathbf{a}\mathbf{r}}$ Generate power ${P}_{1}>0$ until some work $0<{W}_{1}<W$ has been accumulated, i.e. maintain ${P}_{1}$ over the duration $T_{1} : = W_{1} /P_{1}$. Subsequently, maintain the highest possible power $P_{1} > 0$ which allows further work $W_{2} : = W - W_{1}$ to be accumulated, i.e. maintain $P_{2}$ over the duration $T_{2} : = W_{2} /P_{2}$ , where ${P}_{2}$ is chosen such that exhaustion occurs when work $W={W}_{1}+{W}_{2}$ has been accumulated, i.e. at time ${T}_{1}+ {T}_{2}$.

Recall that under the assumptions made in the section “Power–duration models”, we can replace “work” by “distance” and “power” by “velocity”. For instance, when running a 5-km race, the Strategy $\mathbf{P}\mathbf{a}\mathbf{c}{\mathbf{e}}_{\mathbf{c}\mathbf{o}\mathbf{n}}$ implies running continuously at the highest possible *constant* velocity that can be sustained over $D=5000 \ \mathrm{m}$; Strategy $\mathbf{P}\mathbf{a}\mathbf{c}{\mathbf{e}}_{\mathbf{v}\mathbf{a}\mathbf{r}}$ implies running parts of the distance at different velocities, e.g. running the first ${D}_{1}=1000 \ \mathrm{m}$ at some velocity ${V}_{1}$ and then running the remaining ${D}_{2}=D-{D}_{1}=4000 \ \mathrm{m}$ at the velocity ${V}_{2}$ which is chosen such that exhaustion occurs when crossing the finish line.

### Impossibility of over-pacing under the hyperbolic model

Under the hyperbolic model, any pace selection which ensures that (a) ${W}^{\prime}$ is completely depleted by the end of the activity (see subsection “Rate-of-exertion interpretation” in the section "Hyperbolic (a.k.a. critical-power) model"; (b) the power output never drops below $\mathrm{CP}$, is optimal. In principle, athletes could just “burn through” their ${W}^{\prime}$ tank (see subsection “Rate-of-exertion interpretation” in the section "Hyperbolic (a.k.a. critical-power) model" in the first second of the activity and then complete the rest of the activity at $\mathrm{CP}$. In other words, overpacing is impossible in the hyperbolic model; athletes only ever need to worry about “under-pacing” i.e. accidentally dropping the power output below $\mathrm{CP}$. Thus, since $\mathrm{CP}$ is unlikely to be known exactly to the athlete, the hyperbolic model implies that the safest optimal pacing strategy is starting as fast as possible (to avoid the risk of dropping the power output below $\mathrm{CP}$) and then simply “holding on”.

**Fig. 12:**
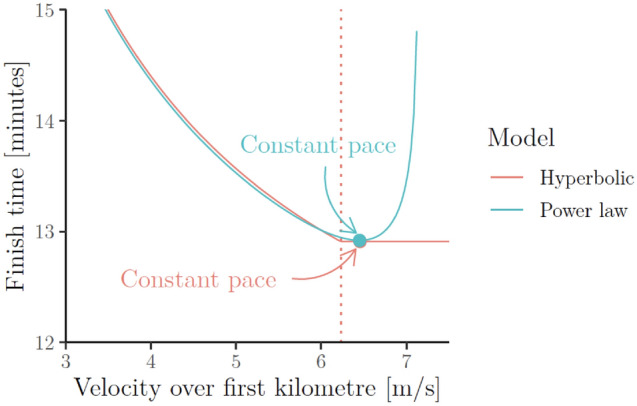
Illustration of Propositions 1 and 2 on a hypothetical 5-km race by Eliud Kipchoge. The lines show Kipchoge’s best possible finish times according to the hyperbolic (a.k.a. critical-power) and power-law (a.k.a. Riegel) model if he maintains the velocity given on the first axis over the first kilometre. The model parameters are as estimated in the section “Case study from Jones et al. (2019)”; the dotted line represents critical velocity. The figure illustrates that the power-law model considers over-pacing to be dangerous whilst the hyperbolic model does not

This result was already proved in Fukuba and Whipp (1999). For completeness, Proposition 1 (proved in Supplementary Information C.1) repeats their result here in the setting described above. It shows that in the hyperbolic model,^10^$\mathbf{P}\mathbf{a}\mathbf{c}{\mathbf{e}}_{\mathbf{c}\mathbf{o}\mathbf{n}}$ and $\mathbf{P}\mathbf{a}\mathbf{c}{\mathbf{e}}_{\mathbf{v}\mathbf{a}\mathbf{r}}$ strategies both lead to the same optimal finishing time as long as ${P}_{1}\ge \mathrm{CP}$, i.e. as long as the athlete does not start out too slowly under $\mathbf{P}\mathbf{a}\mathbf{c}{\mathbf{e}}_{\mathbf{v}\mathbf{a}\mathbf{r}}$. The solid red line in Fig. 12 visualises Proposition 1 in the context of a hypothetical 5-km race (i.e. with power replaced by velocity and work replaced by distance–see “Hyperbolic (a.k.a. critical-power) model”).

#### Proposition 1

(Fukuba and Whipp 1999) *For some work *${W}>{W}^{\prime}$*, let *$T$*, *$P$* as well as *$({P}_{i},{T}_{i},{W}_{i})$* for* $i \in \{\mathrm{1,2}\}$ *be as in Strategies *$\mathbf{P}\mathbf{a}\mathbf{c}{\mathbf{e}}_{\mathbf{c}\mathbf{o}\mathbf{n}}$* and *$\mathbf{P}\mathbf{a}\mathbf{c}{\mathbf{e}}_{\mathbf{v}\mathbf{a}\mathbf{r}}$*, with *${P}_{1}\ne P$*, then:*

1. *if* $${P}_{1}\ge \mathrm{CP}$$ *, then* $${T}_{1}+ {T}_{2}=T$$ *;*
2. *if* $${P}_{1}<\mathrm{CP}$$ *, then* $${T}_{1}+ {T}_{2}>T$$ *.*

### Optimality of even pacing under the power-law model

We now present our main result in this section: Proposition 2 (proved in Supplementary Information C.2) shows that in the power-law model, the “even pacing” strategy, $\mathbf{P}\mathbf{a}\mathbf{c}{\mathbf{e}}_{\mathbf{c}\mathbf{o}\mathbf{n}}$, yields better finishing time than the “uneven pacing” strategy, $\mathbf{P}\mathbf{a}\mathbf{c}{\mathbf{e}}_{\mathbf{v}\mathbf{a}\mathbf{r}}$. That is, pacing is crucial in the power-law model: any deviation from the optimal constant pace leads to an increase in the finishing time (and overpacing is worse than “under-pacing”). The blue line in Fig. 12 visualises the result of Proposition 2. To our knowledge, Proposition 2 and its proof (which uses our new “rate-of-exertion” interpretation of the power-law model presented in subsection “Rate-of-exertion interpretation” of the section "Power-law (a.k.a. Riegel) model") are novel.

#### Proposition 2.

*For some work *$W>0$*, let *$T$*, *$P$* as well as *$({P}_{i},{T}_{i},{W}_{i})$* for* $i \in \{\mathrm{1,2}\}$ *be as in Strategies* $\mathbf{P}\mathbf{a}\mathbf{c}{\mathbf{e}}_{\mathbf{c}\mathbf{o}\mathbf{n}}$ *and *$\mathbf{P}\mathbf{a}\mathbf{c}{\mathbf{e}}_{\mathbf{v}\mathbf{a}\mathbf{r}}$*. Then the finish time under *$\mathbf{P}\mathbf{a}\mathbf{c}{\mathbf{e}}_{\mathbf{v}\mathbf{a}\mathbf{r}}$*, *${T}_{1}+ {T}_{2}$*, grows as the difference between the initial and the optimal even power output, *$\left|{P}_{1}-P\right|$*, increases. In particular, *${T}_{1}+ {T}_{2}>T$* whenever *${ P}_{1}\ne P$*.*

### Pacing results in context

We end this section by putting the pacing implications of the hyperbolic and power-law models in the context of existing research. To ensure that the goal of “winning a race” can be used as a proxy for the goal of “minimising the amount of time needed to generate a fixed amount of work” which we analysed above, we restrict our attention to, essentially, time trials that have standard conditions, e.g. constant course topography and insignificant wind speeds, constant temperature/humidity and the absence of tactics/drafting or other psychological factors that might interact with pacing. We also assume that the race is long enough such that the impact of initial acceleration or the waste of kinetic energy at the end of the race is negligible (de Koning et al. 2011). Under these assumptions, it is thought that the optimal performance in a race is achieved through an even-pacing strategy (Abbiss and Laursen 2008). For instance:

- **Running.** In long-distance running, even pacing is optimal under models based around physics (Keller 1974). Additionally, the previously mentioned online running finish time predictors (e.g. Runner’s World Magazine 2013; Aschwanden 2014) are motivated by the idea an even pace is critical to achieving the best possible performance for a given fitness level. Empirically, faster finishing times are also positively correlated with more even pacing strategies (e.g. Hoffman 2014; Keogh et al. 2020). Finally, even pacing is also recommended by most coaches (e.g. Galloway 2007; Pfitzinger and Douglas 2009) and observed in elite athlete performances. For instance, the 5-km-split times in Eliud Kipchoge’s sub-2-h marathon in 2019 were 00:14:12 ($\pm$ 2 s).
- **Cycling.** In cycling, even pacing strategies are considered to be favourable under the assumptions made above (Foster et al. 1993; de Koning et al. 1999; Atkinson and Brunskill 2000; Atkinson et al. 2003; Ham and Knez 2009); see Coakley and Passfield (2018) for a review and further references. Under the controlled conditions of a 60-min world record attempt, the cyclist in Padilla et al. (2000, Fig. 2) chose a relatively even pace.

We stress again that the assumptions made in this section, and by extension, the optimality of even pacing, may not hold in very short races where the impact of initial acceleration and wasted kinetic energy at the end as well as of other, e.g. physiological or psychological factors, are non-negligible. For instance:

- In a physics-based model from de Koning et al. (1999), fast starts are optimal in 1000 m track cycling events. However, in longer, e.g. 4000 m events, it is optimal to transition to even pacing after a fast start over the first few seconds.
- Bishop et al. (2002) observed that fast starts over the first few seconds (again followed by a transition to constant pace) correlated with improved performance in 2-min kayak ergometer tests.
- Fast starts were observed to correlate with improved performance in 3-min efforts in a study of seven cyclists from Bailey et al. (2011). However, this effect was not observed in 6-min efforts.^11^

In summary, the literature appears to be consistent with the power-law model’s implication that athletes should implement an even pacing strategy (except during the initial acceleration phase which can make up a significant proportion of the effort during short races, e.g. sprints) but not consistent with the hyperbolic model’s implication that athletes never need to worry about over-pacing (all under the assumptions made above).

## Contribution III: Modelling fatigue

**Fig. 13:**
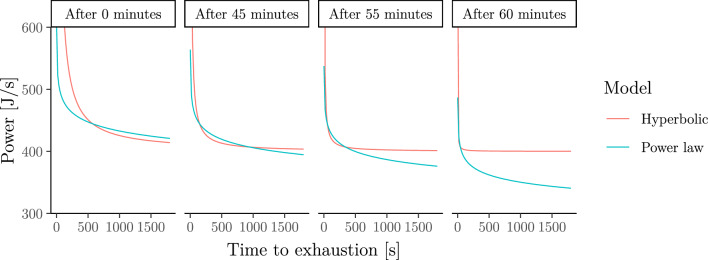
Fatigued power–duration relationship implied by the hyperbolic (a.k.a. critical-power) and power-law (a.k.a. Riegel) models for a cyclist who has already exercised 0, 45, 55 or 60 min at constant power output $P=$ 407 J/s. The figure illustrates the downward shift of the power–duration curve under the power-law model (but not under the hyperbolic model) as the athlete becomes more fatigued

It has been found that the power–duration curve moves downwards as prolonged exercise causes the athlete to fatigue (Leo et al. 2021). This behaviour is not captured by the hyperbolic (a.k.a. critical-power) model as shown in Fig. 13. To circumvent this problem, it has been suggested that the parameter $\mathrm{CP}$ decreases with fatigue (Spragg et al. 2022). In this section, we demonstrate that the power-law (a.k.a. Riegel) model implies a power–duration curve that naturally shifts downwards as the athlete becomes more fatigued without any additional modelling efforts.

### Fatigued power–duration relationship

Assume that an athlete exercises at some constant intensity $P>0$ for some duration $t\ge 0$. If exhaustion has not yet set in at time $t$, then we may be interested in the power–duration relationship of this already partially fatigued athlete. We call this the *fatigued* power–duration relationship. The specific form of the fatigued power–duration relationship depends on the chosen model for the fresh (i.e. non-fatigued) power–duration relationship. Figure 13 illustrates that the hyperbolic and power-law model imply a quite different behaviour of the fatigued power–duration relationship of a cyclist who has already exercised at power output $P=$ 407 [J*/*s] for $t=$ 0, 45, 55 or 60 min. The model parameters are ${W}^{\prime}=\mathrm{25,500}$, $\mathrm{CP}=400$, $F=1.05$, and $S$ is chosen such that the time to exhaustion at power output $P$ is the same in both models. Figure 13 illustrates the following (where $t$ is measured in seconds):

- **Hyperbolic model.** Under the hyperbolic model, the fatigued power–duration relationship again follows a hyperbolic model but with ${W}^{\prime}$ replaced by
  6 $${W}^{\prime}-t\left(P-\mathrm{CP}\right).$$
- **Power-law model.** Under the power-law model, the fatigued power–duration relationship again follows a power-law model but with $S$ replaced by
  7 $${\left({S}^{1/\left(1-E\right)}-t{P}^{1/\left(1-E\right)}\right)}^{1-E}.$$

The result in (6) follows directly from the subsection “Rate-of-exertion interpretation” in the section "Hyperbolic (a.k.a. critical-power) model"; the result in (7) is a consequence of the novel “Rate-of-exertion interpretation” of the power-law model which we introduced in the subsection “Rate-of-exertion interpretation” in the section "Power-law (a.k.a. Riegel) model". More details are given in Supplementary Information D.

### Problems of the “critical power decreases with fatigue” hypothesis

The behaviour of the hyperbolic model shown in Fig. 13 (i.e. the implication that an athlete can always maintain power output equal to at least $\mathrm{CP}$ even when heavily fatigued) contradicts empirical evidence and it has therefore been argued that $\mathrm{CP}$ and ${W}^{\prime}$ should be treated as dynamic quantities that diminish with fatigue (Clark et al. 2019; Spragg et al. 2022). However, assuming that $\mathrm{CP}$ varies with exercise-induced fatigue has two drawbacks.

- **Not inherent in the model.** This assumption requires additional modelling and data-collection efforts.
- **Logical contradiction.** This assumption is also contradictory because if $\mathrm{CP}$ decreases with exercise (specifically: with exercise-induced fatigue) then the power–duration relationship is actually no longer hyperbolic (neither for a fresh nor for a fatigued athlete), i.e. the notion of a “rested” or “fatigued” critical power is not even well defined. To our knowledge, this problem has not been pointed out or addressed in the literature.

In contrast, for the power-law model, Fig. 13 illustrates that the power–duration curve is naturally scaled downwards as the athlete becomes more fatigued. Crucially, this is inherent in the model, i.e. it does not lead to any logical contradictions and no additional data-collection or modelling efforts are needed.

## Conclusions

We have demonstrated, both empirically and theoretically, that the power–duration relationship is more adequately represented by the power-law (a.k.a. Riegel) model than by the hyperbolic (a.k.a. critical-power) model. In particular, our work highlights the following.

1. The power-law model fits power–duration data, e.g. in cycling or rowing, and race results in running better than the hyperbolic model over a wide range of exercise durations or race distances.
2. The power-law model is applicable to a wide range of exercise intensities, durations or distances. In contrast, the hyperbolic is restricted to a very limited range of durations, i.e. between 2 and around 15–25 min.
3. The power-law model only has two parameters, just like the hyperbolic model, and is just as easy to fit.
4. The power-law model appears to be a safer tool for pace selection than the hyperbolic model (except in short sprint races) because it accounts for the fact that over-pacing, e.g. sprinting off on the first kilometre of a long-distance race, is detrimental to the overall performance.
5. The power-law model implies that the power–duration relationship changes with fatigue in a manner that appears to be in closer agreement with empirical evidence than the fatigued power–duration relationship implied by the hyperbolic model.

We stress that we do not claim that the power-law model is “correct”. Indeed, both models likely constitute gross simplifications of reality. For instance, Blythe and Király (2016) found that it can be improved by including additional (individual) correction factors. However, given the choice between the hyperbolic model and the power-law model, Points 1–5 above leave very little reason for choosing the former over the latter for fitness assessment or performance prediction in athletes. The only exception is the modelling of intermittent exercise because, as discussed below, the power-law model cannot easily incorporate recovery.

### Implications for the notion of “critical power”.

It is important to recognise that the term “critical power” (similarly: “critical velocity” or “critical speed”) can refer to two notions which are, in principle, distinct:

- $\mathbf{C}{\mathbf{P}}_{\mathbf{a}\mathbf{s}\mathbf{y}\mathbf{m}\mathbf{p}}$ The power asymptote of the hyperbolic model, i.e. the parameter $\mathrm{CP}$.
- $\mathbf{C}{\mathbf{P}}_{\mathbf{t}\mathbf{h}\mathbf{r}\mathbf{e}\mathbf{s}\mathbf{h}}$ A threshold which is thought to separate distinct physiological responses, and by extension, the “heavy” from the “severe” exercise intensity domain.

Note that critical power in the sense of $\mathbf{C}{\mathbf{P}}_{\mathbf{a}\mathbf{s}\mathbf{y}\mathbf{m}\mathbf{p}}$ only exists because the power–duration relationship is modelled by a hyperbolic function—it does not exist if the data are modelled via a power law which, our work suggests, is the more appropriate functional form. In this precise sense, critical power could be called a “mathematical artefact” as is frequently done by its critics (e.g. Gorostiaga et al. 2022b). The same applies to finite work capacity above critical power, ${W}^{\prime}$. Athletes and coaches should keep this in mind when assessing the meaningfulness of $\mathrm{CP}$ and ${W}^{\prime}$ as metrics for fitness assessment.

We stress that the previous interpretation only concerns $\mathbf{C}{\mathbf{P}}_{\mathbf{a}\mathbf{s}\mathbf{y}\mathbf{m}\mathbf{p}}$. $\mathbf{C}{\mathbf{P}}_{\mathbf{t}\mathbf{h}\mathbf{r}\mathbf{e}\mathbf{s}\mathbf{h}}$, i.e. the notion of critical power as a “physiological threshold” is, in principle, a separate concept. Nonetheless, despite being a physiological threshold, $\mathbf{C}{\mathbf{P}}_{\mathbf{t}\mathbf{h}\mathbf{r}\mathbf{e}\mathbf{s}\mathbf{h}}$ is not easily determined from physiological measurements. Instead, it is commonly argued that $\mathbf{C}{\mathbf{P}}_{\mathbf{t}\mathbf{h}\mathbf{r}\mathbf{e}\mathbf{s}\mathbf{h}}$ coincides with $\mathbf{C}{\mathbf{P}}_{\mathbf{a}\mathbf{s}\mathbf{y}\mathbf{m}\mathbf{p}}$ so that it can be identified by fitting the hyperbolic model to power–duration measurements. Implicit in this practice is the assumption that the changes in physiological responses which separate the “heavy” from the “severe” exercise intensity domain manifest themselves in a “levelling off” of the power–duration curve towards $\mathbf{C}{\mathbf{P}}_{\mathbf{t}\mathbf{h}\mathbf{r}\mathbf{e}\mathbf{s}\mathbf{h}}$ as durations increase towards, say, 15 or 20 min. However, our work suggests that this is not the case. Indeed, if it was the case, the hyperbolic model would fit better than the power-law model for durations up to 15 or 20 min.

### Implications for training prescription

Athletes and coaches often use critical power/velocity (in the sense of $\mathbf{C}{\mathbf{P}}_{\mathbf{a}\mathbf{s}\mathbf{y}\mathbf{m}\mathbf{p}}$) to select training intensities. We stress that setting training intensities is still possible if we instead use the power-law model. In fact, this is consistent with existing practice. For instance:

- In cycling, training intensities are often based on the power that an athlete can sustain for some specific duration (e.g. 60 min in the case of FTP);
- In running, training velocities are often based on the velocity that the runner can sustain over some specific distance (e.g. “5-km pace”, “marathon pace” or “half-marathon pace”).

In both cases, the power-law model gives a straightforward and principled way of estimating the required powers/velocities, e.g. it allows athletes to easily calculate how fast an individual athlete’s “marathon pace” actually is.

### Limitations

The power-law model also has two limitations (though the first is shared by the hyperbolic model):

1. The power-law model assumes that athletes have to come to a complete stop from one second to the next when reaching the limit implied by the power–duration curve (i.e. when their “fatigue” in (5) reaches 1). For instance, the power-law model cannot explain the gradual decrease in power output observed in a three-minute all-out test (Burnley et al. 2006, Fig. 3A) (however, neither can the hyperbolic model). This property may also mean that the penalty for over-pacing shown in Fig. 12 is too harsh.
2. Unlike the hyperbolic model, the power-law model cannot easily be extended to incorporate recovery (such as in Morton and Billat 2004) due to lack of a “critical power” threshold below which recovery can be assumed to occur. We could, of course, easily add a positive constant to (3) as in Tsai (2015) to obtain such a threshold but this would again imply that there exists a non-zero power output which can be sustained for a “very long time without fatigue”.

We are currently developing a new model which retains the advantages of the power-law model whilst resolving both Limitations 1 and 2 (Finke et al. n.d.).

### Supplementary Information

Below is the link to the electronic supplementary material.

Supplementary file1 (PDF 624 KB)

## Data Availability

All data sets used in this manuscript are publicly available. The running data used in “Large-data study in running” is available at the following links https://figshare.com/articles/thepowerof10/3408202, https://figshare.com/articles/Ful_code_to_Prediction_and_Quantification_of_Individual_Athletic_Performance_of_Runners_/3408250. The rowing data set used in “Large-data study in rowing” is available from www.nonathlon.com. The cycling data set used in “Large-data study in cycling” is available from https://github.com/GoldenCheetah/OpenData. Preliminary version of this work has been deposited on bioRxiv https://doi.org/10.1101/2022.08.31.506028.

## Footnotes

1 More precisely, as discussed in the next section, this hyperbolic relationship is assumed to exist for exercise intensities in a limited range which is termed the “severe” intensity domain.

2 A power law was also suggested as an alternative to the hyperbolic model in Housh et al. (1989); Gorostiaga et al. (2022b) but without establishing the link to the existing literature on the power-law model; and, importantly, Gorostiaga et al. (2022b) only used the power-law to model world-record performances *across* different athletes which is different from our goal of modelling the power–duration relationship *within* individual athletes.

3 In cycling, it has been suggested that at least the lower boundary ${P}_{\mathrm{L}}$ can be estimated using a 3-min all-out test (Vanhatalo et al. 2007). However, this test is very arduous on athletes and is known to often overestimate $\mathrm{CP}$ (McClave et al. 2011; Nicolò et al. 2017).

4 As we explain in the subsection “Range of validity” in section "Hyperbolic (a.k.a. critical-power) model" we only fit the hyperbolic model to the 2–15 min range. Supplementary Information B.2 illustrates the compromise in fit when the model is applied to all durations.

5 The problem with the hyperbolic model is *not* that ${P}_{\mathrm{h}\mathrm{y}\mathrm{p}}(T)$ goes to infinity as *T* goes to zero (because this is also the case for ${P}_{\mathrm{p}\mathrm{o}\mathrm{w}}(T)$), but that ${P}_{\mathrm{h}\mathrm{y}\mathrm{p}}\left(T\right)$ goes to infinity *too quickly*.

6 Again, as we explain in the subsection "Range of validity" in section "Hyperbolic (a.k.a. critical-power) model" we only fit the hyperbolic model to the 2–15 min range. Supplementary Information B.3 illustrates the compromise in fit when the model is fit to all durations.

7 Unless it is smoothed out very heavily but then we would end up back with, essentially, a power law.

8 We stress that whilst the data in Fig. 10 exhibit some kinks, e.g. at 3, 7 and 12 min, these should not be taken as evidence of kinks in the underlying power–duration relationship. Rather, most of the other data points likely come from sub-maximal efforts. Indeed, 3, 7 and 12 min are often-recommended test durations for the hyperbolic model (Karsten et al. 2015). Note also that these data were “mined” from training history so that multiple points may correspond to the same activity and then cannot all represent maximal efforts.

9 To simplify the presentation, we consider a variation in pace over only two segments. However, all results remain valid for piecewise-constant pacing strategies with more than two segments.

10 This shortcoming is even worse under a generalisation of the hyperbolic model known as the *three-parameter critical-power* model (Morton 1996) under which an all-out pacing strategy would be *uniquely* optimal as shown in Morton (2009).

11 Fast starts were also observed to correlate with an increased time to exhaustion in a study of another seven cyclists from Jones et al. (2008). However, although fast, slow and even starters accumulated the same amount of work over the initial two minutes, the protocol was not identical for these three conditions afterwards. That is, fast starters may have had a longer time to exhaustion simply because they continued to slow down beyond two minutes whereas the even starters had to keep their (already relatively fast) pace.

## Code availability

The code to produce the figures in this paper is available at the following link (https://github.com/jonahd99/PL_vs_CP).
