# Supplementary material for: Modelling human endurance: power laws vs critical power
Source: Eur J Appl Physiol. 2023 Aug 10;124(2):507–26. doi: 10.1007/s00421-023-05274-5 (PMC10858092; doi:10.1007/s00421-023-05274-5)
Supplement: Supplementary file 1 — Supplementary file1 (PDF 624 KB) [file 421_2023_5274_MOESM1_ESM.pdf]

Supplementary information for "Modelling human endurance: Power laws vs critical power"

Jonah Drake*,‡ Axel Finke*,§ Richard Ferguson†,¶

July 17, 2023

A Additional details for Section 2 in the main manuscript

A.1 Hyperbolic (a.k.a. critical-power) model

A.1.1 Equivalent relationships

Using simple linear algebra, we can verify that the hyperbolic model implies the following relationships which are also illustrated in Figure 1 in the main manuscript. For $W' > 0$, $P > \mathrm{CP} \geq 0$ and $W = PT > W'$:

$$
\begin{aligned}
P &= P_{\text{HYP}}(T) \coloneqq W'/T + \mathrm{CP},\\
P &= P_{\text{HYP}}(W) \coloneqq \mathrm{CP}/(1 - W'/W),\\
T &= T_{\text{HYP}}(P) \coloneqq W'/(P - \mathrm{CP}),\\
T &= T_{\text{HYP}}(W) \coloneqq (W - W')/\mathrm{CP},\\
W &= W_{\text{HYP}}(T) \coloneqq W' + \mathrm{CP} \cdot T,\\
W &= W_{\text{HYP}}(P) \coloneqq W'/(1 - \mathrm{CP}/P).
\end{aligned}
$$

As discussed in Section 2.1 in the main manuscript, we sometimes replace "power" (P) by "velocity" (V) and "work" (W) by "distance" (D). In this case, we also write V_{HYP} and D_{HYP} instead of P_{HYP} and W_{HYP}.

*Department of Mathematical Sciences, School of Science, Loughborough University, UK
†School of Sport, Exercise and Health Sciences, Loughborough University, UK
‡orcid.org/0000-0003-2463-350X, j.p.drake@lboro.ac.uk
§orcid.org/0000-0002-8379-3012
¶orcid.org/0000-0002-2508-8358

A.2 Power-law (a.k.a. Riegel) model

A.2.1 Equivalent relationships

Using simple linear algebra, we can again verify that the power-law model implies the following relationships which are also illustrated in Figure 2 in the main manuscript. For $S > 0$, $F = 1/E > 1$ and $W = P \cdot T > 0$:

$$\begin{aligned}
P &= P_{\text{POW}}(T) \coloneqq S \cdot T^{E-1},\\
P &= P_{\text{POW}}(W) \coloneqq S^F W^{1-F},\\
T &= T_{\text{POW}}(P) \coloneqq (S/P)^{1/(1-E)},\\
T &= T_{\text{POW}}(W) \coloneqq (W/S)^F,\\
W &= W_{\text{POW}}(T) \coloneqq S \cdot T^E,\\
W &= W_{\text{POW}}(P) \coloneqq (S^F/P)^{1/(F-1)}.
\end{aligned}$$

As discussed in Section 2.2.3 in the main manuscript, we sometimes replace "power" (P) by "velocity" (V) and "work" (W) by "distance" (D). In this case, we also write V_{POW} and D_{POW} instead of P_{POW} and W_{POW}.

A.2.2 Bias–variance trade-off and Riegel-predictors

Recall that, as mentioned in Section 2.2.3 in the main manuscript, sufficiently many data points are needed to accurately estimate the speed parameter, S, and endurance parameter, E, (equivalently: the fatigue factor $F = 1/E$) of the power-law model.

In practice, there is often a need to form predictions based on a very small number of data points – possibly just a single observation, e.g. when predicting a marathon finish time from a single prior half-marathon result.

In this case, it is common to fix E (equivalently: $F = 1/E$) to a suitable default value which then permits the estimation of S from a single data point. Assume that we have a previous power measurement P_0 recorded over some duration T_0. Then solving Equation 3 from the main manuscript for S and using (P_0, T_0) in place of (P, T), gives the estimate $S \approx \widehat{S} \coloneqq P_0 T_0^{1-E}$; and plugging $\widehat{S}$ back into Equation 3 from the main manuscript implies the power–duration relationship:

$$P = \widehat{S} T^{1/F-1} = P_0 \left(\frac{T}{T_0}\right)^{E-1}. \tag{1}$$

When applied to, e.g., running, this gives an easy way of predicting the finish time T in a race over distance D from the finish time T_0 in a previous race over some other distance D_0. More specifically, after replacing power P by velocity $V = D/T$ (see Section 2.1 in the main manuscript) and E by $1/F$, we can re-arrange (1) as

$$T = T_0 \left(\frac{D}{D_0}\right)^{F}. \tag{2}$$

For instance, the finish-time calculator from Runner's World Magazine (2013) implements (2) with $F = 1.06$ for all runners. This comes with the assumption that the user is an average runner with without "a natural significant bias towards either speed or endurance" (Runner's World Magazine, 2013).

Of course, the fatigue factor F (equivalently: the endurance parameter $E = 1/F$) is likely different for each person as shown in Blythe and Király (2016); Zinoubi et al. (2017). Hence, setting F (equivalently: E) to a default value incurs a bias. However, this bias can sometimes be outweighed by the variance reductions brought about by avoiding the need for estimating F from limited amounts of data. Thus, fixing the fatigue factor to a sensible default value may sometimes be justifiable as a *bias–variance trade-off*.

B Additional details for Section 3 in the main manuscript

B.1 Model behaviour over short durations

The hyperbolic (a.k.a. critical-power) model implies that any sufficiently small amount of work $0 < W < W'$ can be generated arbitrarily quickly. For instance, if the model was applied to short durations, it would thus imply that an elite runner can "teleport" over more than one hundred metres. In contrast, power-law (a.k.a. Riegel) model has no such unrealistic implication. Informally, these results can be seen from Figures 1c and 2c in the main manuscript which show that the second-axis intercept of the work–duration curve is

- $W' > 0$ under the hyperbolic model;
- 0 under the power-law model.

More formal proofs – which are needed because the power–duration relationship $P(T)$ is not actually well defined at $T = 0$ – are given in Propositions 3 and 4.

Proposition 1. *Let $0 < W < W'$. Then for any $T > 0$, $W < T \cdot P_{\text{HYP}}(T)$.*

Proof. Let $T > 0$. Then since $W < W'$,

$$T \cdot P_{\text{HYP}}(T) = W' + T \cdot \text{CP} > W + T \cdot \text{CP} > W.$$

This completes the proof. □

Proposition 2. *Let $S > 0$ and $F > 1$. Then for any $W > 0$, there exists a unique duration $T > 0$ such that $W = T \cdot P_{\text{POW}}(T)$.*

Proof. The function $h\colon [0, \infty) \to [0, \infty)$, $h(T) \coloneqq T \cdot P_{\text{POW}}(T) = S \cdot T^{1/F}$ is strictly increasing and $h(0) = 0$. Thus, there exists a unique value $T > 0$ such that $W = h(T) = T \cdot P_{\text{POW}}(T)$. □

B.2 Case study from Jones et al. (2019)

Table 1 shows Eliud Kipchoge's personal records in different running events (as of 5 November 2021). The data were collected from World Athletics (2021). Note that the difference between, e.g., the 5000 m and 5 km event is that the former was run on the track whereas the latter was run on the road.

Table 1: Personal records of Eliud Kipchoge in different events.

Event	Time [h:min:s]	Velocity [m/s]
1500 m	00:03:33	7.04
1500 m (indoor)	00:03:36	6.94
One mile	00:03:50	6.98
3000 m	00:07:28	6.70
3000 m (indoor)	00:07:29	6.68
Two miles	00:08:08	6.60
Two miles (indoor)	00:08:07	6.60
5000 m	00:12:47	6.52
5000 m (indoor)	00:12:56	6.45
10 000 m	00:26:49	6.21
5 km	00:13:11	6.32
10 km	00:28:11	5.91
Half marathon	00:59:25	5.92
30 km	01:27:13	5.73
Marathon	02:01:39	5.78

We now provide a version of Figure 4 from the main manuscript in which we fit the hyperbolic model to all personal records up to marathon distance in the same way as the power-law model.

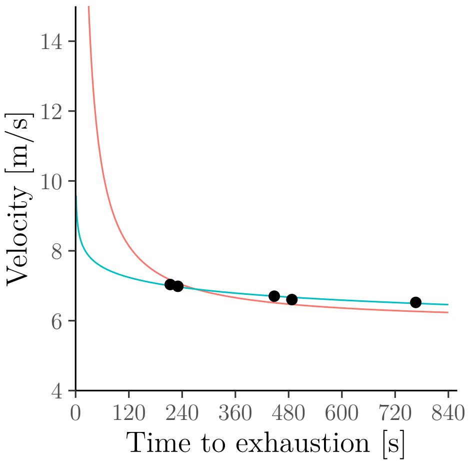

(a) Velocity vs duration (2–15 minutes).

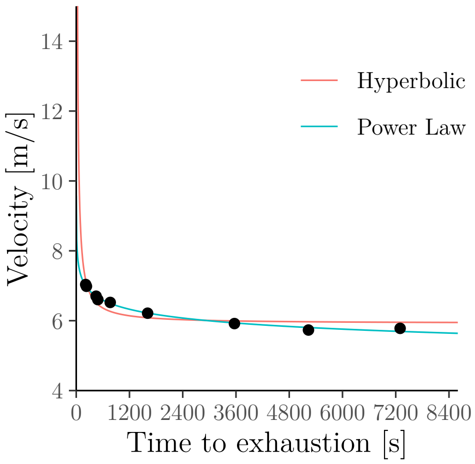

(b) Velocity vs duration.

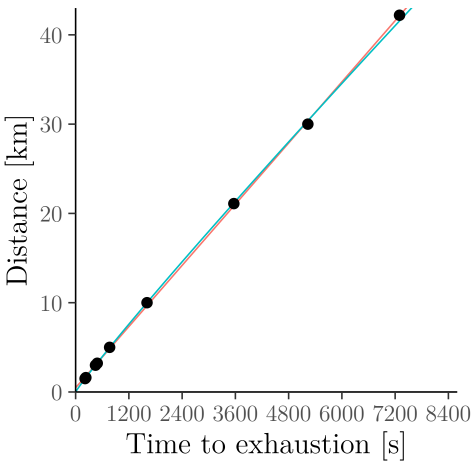

(c) Distance vs duration.

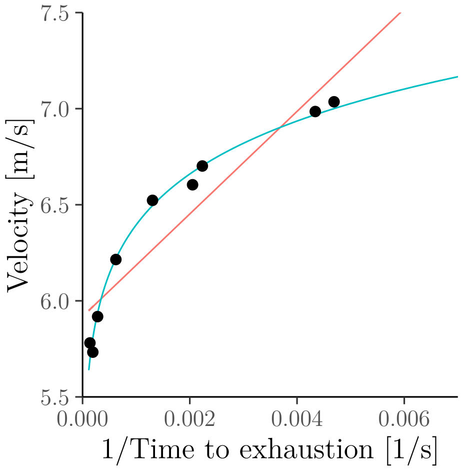

(d) Velocity vs reciprocal duration.

Figure 1: A reproduction of Figure 4 from the main manuscript but the hyperbolic (a.k.a. critical power) model is now fitted to *all* available data. This reduces the error for shorter and longer durations but increases the error in the 2–15 minute range.

B.3 Case study from Jones and Vanhatalo (2017)

Table 2 shows Haile Gebrselassie's personal records in different running events. The data were collected from World Athletics (2021). Note that the difference between, e.g., the 10 000 m and 10 km event is that the former was run on the track whereas the latter was run on the road.

Table 2: Personal records of Haile Gebrselassie in different events.

Event	Time [h:min:s]	Velocity [m/s]
800 m (indoor)	00:01:49	7.32
1500 m (indoor)	00:03:34	7.02
1500 m (indoor)	00:03:32	7.08
One mile	00:03:52	6.93
2000 m (indoor)	00:04:53	6.83
3000 m	00:07:25	6.74
3000 m (indoor)	00:07:26	6.72
Two miles	00:08:01	6.69
Two miles (indoor)	00:08:05	6.64
5000 m	00:12:39	6.58
5000 m (indoor)	00:12:50	6.49
10 000 m	00:26:23	6.32
20 000 m	00:56:26	5.91
One hour	01:00:00	5.91
10 km	00:27:02	6.17
15 km	00:41:38	6.00
10 miles road	00:44:24	6.04
20 km	00:55:48	5.97
Half marathon	00:58:55	5.97
25 km	01:11:37	5.82
Marathon	02:03:59	5.67

We now provide a version of Figure 5 from the main manuscript in which we fit the hyperbolic model to all personal records up to marathon distance in the same way as the power-law model.

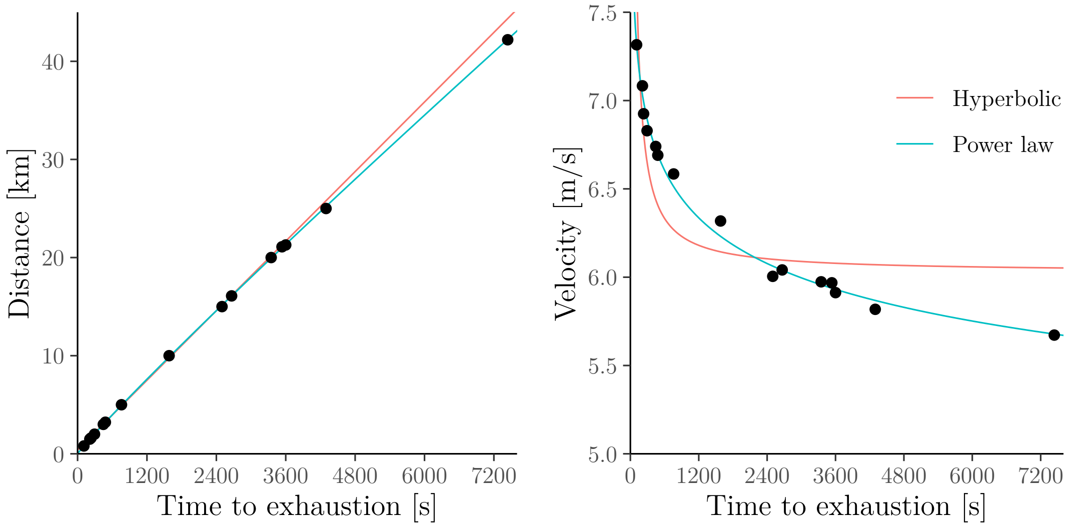

(a) Distance vs duration.

(b) Velocity vs duration.

Figure 2: A reproduction of Figure 5 from the main manuscript but the hyperbolic (a.k.a. critical power) model is now fitted to *all* available data. This reduces the error for shorter and longer durations but increases the error in the 1500–15 000 m range.

We now provide a version of Figure 6 from the main manuscript in which we fit the hyperbolic model to all personal records up to marathon distance in the same way as the power-law model.

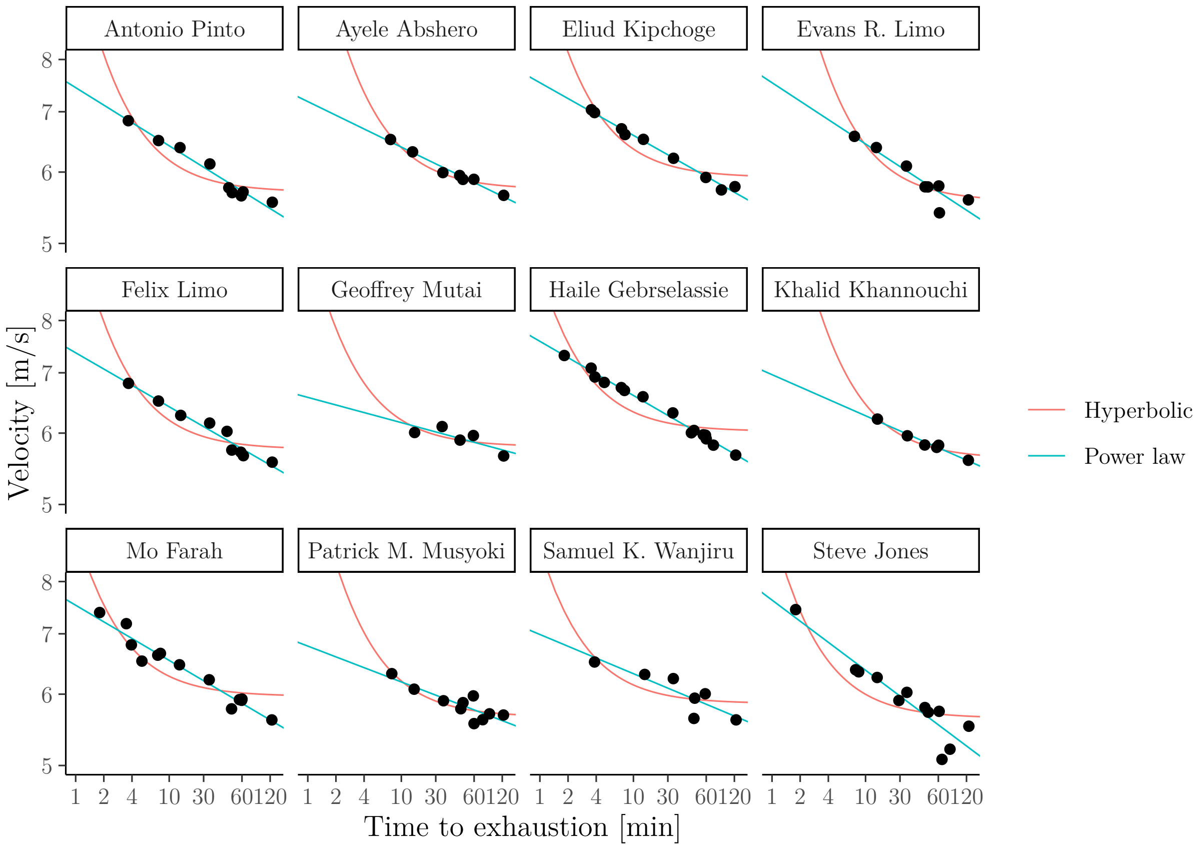

Figure 3: A reproduction of Figure 6 from the main manuscript but the hyperbolic (a.k.a. critical power) model is now fitted to *all* available data. This reduces the error for shorter and longer durations but increases the error in the 1500–15 000 m range.

Finally, we provide a numerical comparison of the relative errors of both models for the athletes used in Section 3.2 in the main manuscript.

Table 3: Relative errors of the hyperbolic (a.k.a. critical-power) and power-law (a.k.a. Riegel) models for the 12 athletes from Jones and Vanhatalo (2017) fitted either to personal records over 1500–15 000 m or all available personal records (up to marathon distance). Errors are calculated as the relative distance between observed and predicted velocities as explained in Section B.4. Colours highlight the better-performing model.

	1500–15 000 m		0–42 195 m	
Athlete	Hyperbolic	Power law	Hyperbolic	Power law
Antonio Pinto	2.1700	**1.0000**	2.6900	**1.1200**
Ayele Abshero	0.5700	**0.4700**	0.9700	**0.5600**
Eliud Kipchoge	0.8400	**0.3500**	2.5000	**0.6000**
Evans Rutto Limo	1.6600	**0.7600**	2.0300	**1.6900**
Felix Limo	0.7500	**0.5600**	2.8100	**1.1300**
Geoffrey Mutai	1.2200	**1.1800**	1.7100	**1.3900**
Haile Gebrselassie	1.5100	**0.7900**	3.2300	**0.5700**
Khalid Khannouchi	0.2900	**0.0400**	0.6800	**0.4400**
Mo Farah	1.7200	**1.4300**	3.1500	**1.4500**
Patrick Makau Musyoki	**0.3400**	0.3500	**1.2900**	1.5000
Samuel Kamau Wanjiru	3.3900	**2.6500**	3.0200	**1.9800**
Steve Jones	1.1200	**0.7500**	4.0900	**2.8400**
Average	1.2983	**0.8608**	2.3417	**1.2667**

B.4 Model-error computation

Here, we explain more formally how the model errors in Sections 3.3, 3.4, and 3.5 in the main manuscript and in Table 3 from Section B.3 are calculated.

Assume that (after cleaning), our data set contains N athletes and that $K_n \geq 2$ pairs of power and duration (or velocity and duration, in the case of running) measurements $\{(P_n^k, T_n^k) \mid 1 \leq k \leq K_n\}$ are available for the nth athlete. Then the average relative error of the hyperbolic model for durations between T_0 and T_1 is calculated as follows.

1. Let (CP_n, W_n') be the estimate of (CP, W') obtained via linear regression using only data corresponding to activities whose duration is no shorter than T_0 and no longer than T_1, i.e. using only the measurements $\{(P_n^k, T_n^k) \mid k \in \mathcal{K}_n\}$, where $\mathcal{K}_n := \{k \in \{1, \dots, K_n\} \mid T_0 \leq T_n^k \leq T_1\}$.

2. Let $\widehat{P}_n^k := W_n'/T_n^k + \mathrm{CP}_n$ be the fitted power for the kth activity of the nth athlete under the hyperbolic model. The relative error of the hyperbolic model for the data from the nth athlete in this exercise duration range is then given by

$$\mathrm{error}_n := \frac{1}{\#\mathcal{K}_n} \sum_{k \in \mathcal{K}_n} \left| \frac{P_n^k - \widehat{P}_n^k}{P_n^k} \right|,$$

where $\#\mathcal{K}_n$ is the cardinality of $\mathcal{K}_n$ (i.e. the number of power–duration measurements for the nth athlete in this exercise duration range).

3. Then the average relative error of the hyperbolic model for this exercise duration range (i.e. the height of one of the red bars in Figures 7, 8 or 9 from the main manuscript) is then given by the following weighted average (where we weight by the number of available data points for each athlete):

$$\text{error} \coloneqq \sum_{n=1}^{N} w_n \text{error}_n.$$

where we have defined the nth weight as:

$$w_n \coloneqq \frac{\#\mathcal{K}_n}{\sum_{m=1}^{N} \#\mathcal{K}_m}.$$

4. The associated standard error (i.e. the size of the error bar in Figures 7, 8 or 9 from the main manuscript) is then calculated as:

$$\text{se} \coloneqq \sqrt{\text{var} \sum_{n=1}^{N} w_n^2},$$

where

$$\text{var} \coloneqq \frac{1}{N} \sum_{n=1}^{N} \left[\text{error}_n - \left(\frac{1}{N} \sum_{m=1}^{N} \text{error}_m \right) \right]^2.$$

That is, if all weights are equal to $1/N$, then $\text{se} = \sqrt{\text{var}/N}$ reduces to the usual standard error of the (unweighted) mean.

For the power-law model, we proceed analogously. The only difference is that we instead compute linear-regression estimates (S_n, E_n) of (S, E) and then define the fitted values as $\widehat{P}_n^k \coloneqq S_n (T_n^k)^{E_n - 1}$.

B.5 Large-data study in running

Here, we give additional details about the data set used for the study in Section 3.3 in the main manuscript. The data were collected by Blythe and Király (2016) and are available for download here: `https://figshare.com/articles/dataset/thepowerof10/3408202`. For the study, we removed all runners who have at most three race results with finishing times in the 2–15 minute range or at most two race results with finishing times in the 2–15 minute range over distinct distances.

B.6 Large-data study in rowing

Here, we give additional details about the data set used for the study in Section 3.4 in the main manuscript. The data were collected on 28 August 2022 from `www.nonathlon.com` for the years (i.e. seasons) from 2002 to 2022. The data are given either as finish times T over fixed distances D (500 m, 1 km, 5 km, 6 km, 10 km, 21.0975 km and 42.195 km) or as covered distances D over fixed durations (30 min and 60 min). To calculate the power output P, we use the commonly used conversion, where T is in seconds, D is in metres and P is in joules per second:

$$P = 2.8\left(\frac{D}{T}\right)^3.$$

Finally, we removed all athlete seasons which have less than three records in the 2–20 minute range, and any athlete season which reported an (equivalent) average power output of over 1300 J/s leaving 3244 unique athlete seasons.

B.7 Large-data study in cycling

We downloaded all the possible data (as of 9 June 2022) from Golden Cheetah using the GoldenCheetahOpenData (v0.2; Kosmidis, 2022) package in R (v4.1.2; R Core Team, 2021). We created a data set consisting of, for each athlete n, the highest measured average power outputs, $\mathrm{MMP}_{n,x}$, over given durations, $x > 0$. These are often referred to as *mean maximal power (MMP)* values. Before fitting the models, we excluded some data points. We now give more details.

- Power-meter malfunctions are not uncommon and can overestimate the power being produced by an athlete. To alleviate this problem, we removed rides which would have led to unrealistically high MMP values. This is done as follows, where N is the number of athletes and where x takes values in a set of 54 durations X, ranging from five seconds to two hours.
  1. Calculate "auxiliary" MMP values $\widetilde{\mathrm{MMP}}_{n,x}$ for each athlete n and duration x based on the original data set.
  2. Calculate the 95th percentile q_x of $\widetilde{\mathrm{MMP}}_{1,x}, \dots, \widetilde{\mathrm{MMP}}_{N,x}$.
  3. Discard any ride of any athlete n which contains an auxiliary MMP value which is such that $\widetilde{\mathrm{MMP}}_{n,x} > q_x$.
  4. For each athlete n and each duration x, compute the MMP values $\mathrm{MMP}_{n,x}$ based on the remaining rides.
- Many of the MMP values calculated as above likely correspond to submaximal efforts which should not be used to estimate the models. Unfortunately, it is unknowable for which of these values this is the case. However, values $\mathrm{MMP}_{n,x}$ such that $\mathrm{MMP}_{n,x} \leq \mathrm{MMP}_{n,y}$, for some $x < y$, *cannot* be maximal and were therefore excluded. We stress that this does not necessarily mean that all the remaining data points correspond to maximal efforts.

- We removed athletes with fewer than three MMP values in the 2–15 minute range. This is because both models would have zero error if fitted to only two data points.

B.8 Piecewise-defined models

In this section, we provide details on the "piecewise-defined" models from Péronnet and Thibault (1989); Puchowicz et al. (2020); Luttikholt and Jones (2022). Throughout, $T_* > 0$ denotes the threshold which is such that the power–duration relationship $P(T)$ is (approximately) hyperbolic for durations $T \leq T_*$ whilst a different functional form is used for durations $T > T_*$. Furthermore, we let $\mathbb{I}$ denote the indicator function, i.e. $\mathbb{I}\{T > T_*\} = 1$ if $T > T_*$ and $\mathbb{I}\{T > T_*\} = 0$, otherwise.

B.8.1 Péronnet and Thibault (1989)

Let $T_* = 420\,\mathrm{s}$ (7 min) be the threshold parameter (denoted T_{MAP} in Péronnet and Thibault 1989) and let $k_1 = 30$, $k_2 = 20$, $f = -0.233$, and $\mathrm{BMR} = 1.2$ be known constants. Furthermore, let $A, \mathrm{MAP} > 0$ and $E < 0$ be other unknown (and athlete-specific) model parameters. Then the model from Péronnet and Thibault (1989) is

$$P(T) = \frac{A(1 - \mathrm{e}^{-T/k_2}) - k_1(\mathrm{MAP} - \mathrm{BMR})(1 - \mathrm{e}^{-T/k_1})}{T} + \mathrm{MAP} \\ + \mathbb{I}\{T > T_*\} \log(T/T_{\mathrm{MAP}}) \left(\frac{Af(1 - \mathrm{e}^{-T/k_2}) - k_1 E(1 - \mathrm{e}^{-T/k_1})}{T} + E \right).$$

Figure 4 illustrates the model and shows that it has a "kink" in the power–duration curve at duration $T = T_*$. Note that $\lim_{T \to \infty} P(T) = -\infty$.

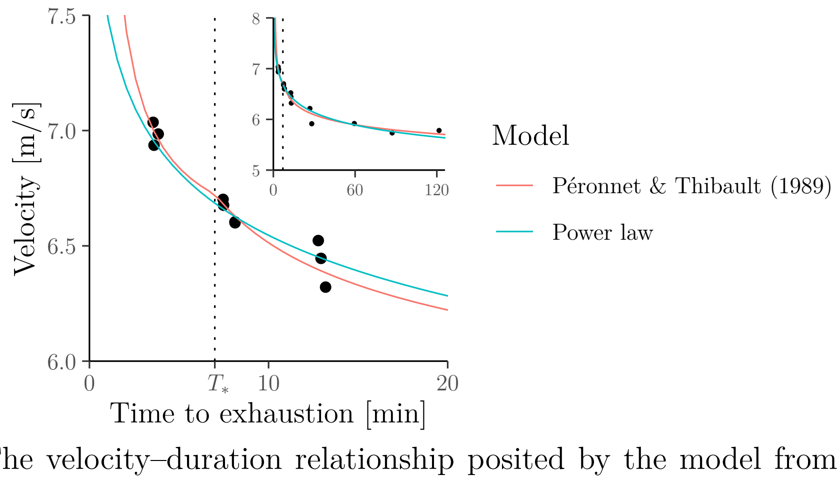

Figure 4: The velocity–duration relationship posited by the model from Péronnet and Thibault (1989) fitted to Eliud Kipchoge's personal records.

B.8.2 Puchowicz et al. (2020)

Let $T_* = 1800\,\mathrm{s}$ (30 min) be the threshold parameter (denoted TCPmax in Puchowicz et al. 2020) and let $W', \mathrm{CP}, P_{\mathrm{MAX}}, A > 0$ be unknown athlete-specific parameters.

Puchowicz et al. (2020) propose the following three models.

- The *OmPD* model is given by

$$P(T) = \frac{W'(1 - \mathrm{e}^{-T(P_{\text{MAX}} - \mathrm{CP})/W'})}{T} + \mathrm{CP} - \mathbb{I}\{T > T_*\}A \log(T/T_*).$$

- The *Om3CP* model is given by

$$P(T) = \frac{W'}{T + W'/(P_{\text{MAX}} - \mathrm{CP})} + \mathrm{CP} - \mathbb{I}\{T > T_*\}A \log(T/T_*).$$

- The *OmExp* model is given by

$$P(T) = (P_{\text{MAX}} - \mathrm{CP})\mathrm{e}^{-T(P_{\text{MAX}} - \mathrm{CP})/(\mathrm{e}W')} + \mathrm{CP} - \mathbb{I}\{T > T_*\}A \log(T/T_*).$$

Figure 10 in the main manuscript illustrates these models and shows that they again have a "kink" in the power–duration curve at duration $T = T_*$. Additionally, for all of these models, $\lim_{T\to\infty} P(T) = -\infty$.

B.8.3 Luttikholt and Jones (2022)

Let $T_* = 360\,\mathrm{s}$ ($6\,\mathrm{min}$) be the threshold parameter and let $W', \mathrm{CP} > 0$ and $0 < E < 1$ be athlete-specific parameters. Then the model from Luttikholt and Jones (2022) uses the hyperbolic (a.k.a. critical-power) model for durations up to T_* and uses a power-law model for durations longer than T_*, i.e.:

$$P(T) = \begin{cases} \dfrac{W'}{T} + \mathrm{CP}, & \text{if } T \leq T_*, \\ ST^{E-1}, & \text{if } T > T_*, \end{cases}$$

where continuity of the power–duration curve is ensured by setting

$$S \coloneqq W'/T_*^E + \mathrm{CP}/T_*^{E-1}.$$

Figure 5 illustrates the model and shows that it again has a "kink" in the power–duration curve at duration $T = T_*$. However, this kink is not very pronounced due to the small value of the threshold T_*, i.e. because the hyperbolic model is only used for durations up to 6 minutes here.

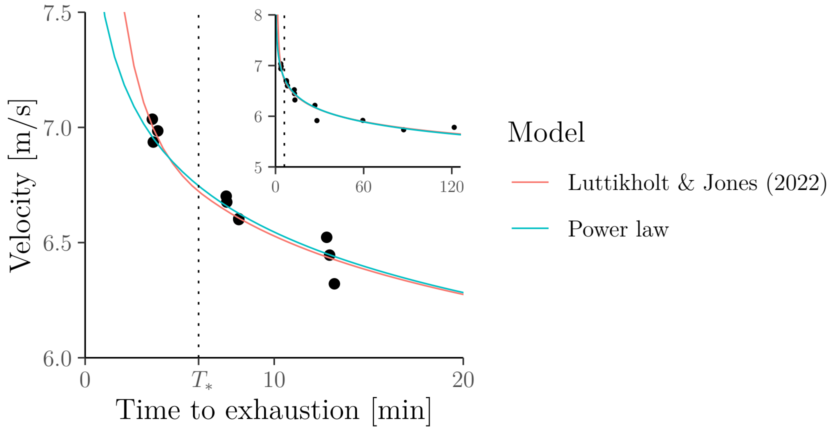

Figure 5: The velocity–duration relationship posited by the model from Luttikholt and Jones (2022) fitted to Eliud Kipchoge's personal records.

C Additional details for Section 4 in the main manuscript

C.1 Impossibility of overpacing under the hyperbolic model

Proof (of Proposition 1). Since Strategy Pace_{con} leads to exhaustion at time T at which point work W has been accumulated, we must have

$$T = T_{\text{HYP}}(W) = (W - W')/\text{CP}.$$

Likewise, since Strategy Pace_{var} leads to exhaustion at time $T_1 + T_2$ at which point work W has been accumulated, we must have the identities:

$$P_2 = (W - T_1 P_1)/T_2, \quad (3)$$

$$W' - (P_1 - \text{CP})_+ T_1 - (P_2 - \text{CP})T_2 = 0, \quad (4)$$

where $x_+ := \max\{x, 0\}$ and where we recall that $T_1 = W_1/P_1$. Substituting (3) in (4) and solving for T_2 then gives

$$T_2 = \begin{cases} (W - W')/\text{CP} - T_1, & \text{if } P_1 \geq \text{CP}, \\ (W - T_1 P_1 - W')/\text{CP}, & \text{if } P_1 < \text{CP}. \end{cases}$$

We are now ready to prove Part 1. If $P_1 \geq \text{CP}$, then

$$T_1 + T_2 = (W - W')/\text{CP} = T.$$

We now prove Part 2. If $P_1 < \text{CP}$, then

$$T_1 + T_2 = (W - T_1 P_1 - W')/\text{CP} + T_1 = T + T_1(1 - P_1/\text{CP}) > T.$$

This completes the proof. □

C.2 Optimality of even pacing under the power-law model

Proof (of Proposition 2). Since Strategy Pace$_{\text{con}}$ leads to exhaustion when work W has been accumulated, we must have

$$P = P_{\text{POW}}(W) = (S/W^{1-1/F})^F.$$

Likewise, since Strategy Pace$_{\text{var}}$ leads to exhaustion at time $T_1 + T_2$ at which point work W has been accumulated, we must have the identities:

$$P_2 = (W - T_1 P_1)/T_2, \tag{5}$$
$$S^{F/(F-1)} - P_1^{F/(F-1)} T_1 - P_2^{F/(F-1)} T_2 = 0, \tag{6}$$

where we recall that $T_1 = W_1/P_1$ and where (6) follows from our novel "rate-of-exertion" interpretation in Equation 4 and 5 from the main manuscript because, noting that $1/(1-E) = F/(F-1)$:

$$\begin{aligned}\text{Fatigue}_{T_1+T_2} = 1 &\iff \text{rate}_{\text{POW}}(P_1)T_1 + \text{rate}_{\text{POW}}(P_2)T_2 = 1\\ &\iff P_1^{F/(F-1)}T_1 + P_2^{F/(F-1)}T_2 = S^{F/(F-1)}.\end{aligned}$$

Substituting (5) in (6) and solving for T_2 (now interpreted as a function of P_1) then gives

$$T_2 = T_2(P_1) = \left[\frac{W - T_1 P_1}{(S^{F/(F-1)} - P_1^{F/(F-1)} T_1)^{1-1/F}}\right]^F.$$

It remains to show that $\tilde{T}(P_1) := T_1(P_1) + T_2(P_1) = W_1/P_1 + T_2(P_1)$ has a minimum at $P_1 = P$. We have

$$\frac{\partial}{\partial P_1}\tilde{T}(P_1) = g(P_1)h(P_1),$$

where

$$h(P_1) := \left(\frac{W - W_1}{(P/P_1)^{1/(F-1)} W - W_1}\right)^F - 1,$$

for some function $P_1 \mapsto g(P_1)$ which is strictly positive on $(0, P_{\text{POW}}(W_1))$ for $S > 0$, $F > 1$ and $0 < W_1 < W$ (to keep the notation simple, we do not make the dependence of g and h on W_1, W, S and F explicit in the notation). The proof is then complete since $h((1+x)P)$ is increasing in x and $h(P) = 0$; in other words, $\tilde{T} = T_1 + T_2$ has a unique minimum at $P_1 = P$. This completes the proof. □

D Additional details for Section 5 in the main manuscript

Here, we provide the mathematical details for the fatigued power–duration relationships illustrated in Figure 13 from the main manuscript. Assume that the athlete has already

exercised over some duration $t > 0$ (starting from a fully rested state). Specifically, let $P\langle s\rangle$ be the instantaneous power output generated by the athlete at time $0 \leq s \leq t$. For instance, in the numerical example from Section 5.1 of the main manuscript, we have that $P\langle s\rangle = P$ for any $s > 0$.

The specific form of the fatigued power–duration relationship depends on the chosen model for the fresh (i.e. non-fatigued) power–duration relationship.

- **Hyperbolic model.** If the fresh power–duration relationship is described by the hyperbolic model, and assuming for simplicity that $P\langle s\rangle \geq \mathrm{CP}$ for all $0 \leq s \leq t$, then the fatigued power–duration relationship is again a hyperbolic model but with W' replaced by $W' - \int_0^t (P\langle s\rangle - \mathrm{CP})\,\mathrm{d}s$.

- **Power-law model.** If the fresh power–duration relationship is described by the power-law model, the fatigued power–duration relationship is again a power-law model but with S replaced by $(S^{1/(1-E)} - \int_0^t P\langle s\rangle^{1/(1-E)}\,\mathrm{d}s)^{1-E}$.

The first result follows directly from Section 2.1.4 in the main manuscript; the second result is a consequence of the novel "rate-of-exertion interpretation" of the power-law model which we introduced in Section 2.2.4 in the main manuscript.

References

Blythe, D. A. and Király, F. J. (2016). Prediction and quantification of individual athletic performance of runners. *PLoS One*, 11(6):1–16.

Jones, A. M., Burnley, M., Black, M. I., Poole, D. C., and Vanhatalo, A. (2019). The maximal metabolic steady state: redefining the 'gold standard'. *Physiological Reports*, 7(10):e14098.

Jones, A. M. and Vanhatalo, A. (2017). The 'critical power' concept: Applications to sports performance with a focus on intermittent high-intensity exercise. *Sports Medicine*, 47(1):65–78.

Kosmidis, I. (2022). *GoldenCheetahOpenData: R API and Local Database Management for the GoldenCheetah OpenData Project*. R package version 0.2.

Luttikholt, H. and Jones, A. M. (2022). Effect of protocol on peak power output in continuous incremental cycle exercise tests. *European Journal of Applied Physiology*, 122(3):757–768.

Péronnet, F. and Thibault, G. (1989). Mathematical analysis of running performance and world running records. *Journal of Applied Physiology*, 67(1):453–465.

Puchowicz, M. J., Baker, J., and Clarke, D. C. (2020). Development and field validation of an omni-domain power-duration model. *Journal of Sports Sciences*, 38(7):801–813.

R Core Team (2021). *R: A Language and Environment for Statistical Computing*. R Foundation for Statistical Computing, Vienna, Austria.

Runner's World Magazine (2013). RW's race time predictor. https://www.runnersworld.com/uk/training/a761681/rws-race-time-predictor/. Accessed: 2021-12-13.

World Athletics (2021). Athletes hub. https://www.worldathletics.org/athletes. Accessed: 2021-11-05.

Zinoubi, B., Vandewalle, H., and Driss, T. (2017). Modeling of running performances in humans: Comparison of power laws and critical speed. *The Journal of Strength & Conditioning Research*, 31(7):1859–1867.
